# Achieving of high-diet-fiber barley via managing fructan hydrolysis

**DOI:** 10.1038/s41598-022-21955-1

**Published:** 2022-11-09

**Authors:** Mingliang Fei, Yunkai Jin, Jia Hu, Gleb Dotsenko, Ying Ruan, Chunlin Liu, Gulaim Seisenbaeva, Annica A. M. Andersson, Roger Andersson, Chuanxin Sun

**Affiliations:** 1 Key Laboratory of Crop Epigenetic Regulation and Development in Hunan Province, Hunan Agricultural University, Changsha, 410128 China; 2 Department of Plant Biology, Uppsala BioCenter, Linnean Centre for Plant Biology, Swedish University of Agricultural Sciences (SLU), P.O. Box 7080, 750 07 Uppsala, Sweden; 3 Key Laboratory of Education Department of Hunan Province On Plant Genetics and Molecular Biology, College of Bioscience and Biotechnology, Hunan Agricultural University, Changsha, 410128 China; 4 Department of Molecular Sciences, Uppsala BioCenter, Swedish University of Agricultural Sciences, P.O. Box 7015, 750 07 Uppsala, Sweden; 5 College of Agronomy, Hunan Agricultural University, Changsha, 410128 China

**Keywords:** Biological techniques, Molecular biology, Plant sciences

## Abstract

High fructan content in the grain of cereals is an important trait in agriculture such as environmental resilience and dietary fiber food production. To understand the mechanism in determining final grain fructan content and achieve high fructan cereal, a cross breeding strategy based on fructan synthesis and hydrolysis activities was set up and have achieved barley lines with 11.8% storage fructan in the harvested grain. Our study discovered that high activity of fructan hydrolysis at later grain developmental stage leads to the low fructan content in mature seeds, simultaneously increasing fructan synthesis at early stage and decreasing fructan hydrolysis at later stage through crossing breeding is an efficient way to elevate grain diet-fiber content. A good correlation between fructan and beta glucans was also discovered with obvious interest. Field trials showed that the achieved high fructan barley produced over seven folds higher fructan content than control barley and pull carbon-flux to fructan through decreasing fructan hydrolysis without disruption starch synthesis will probably not bring yield deficiency.

## Introduction

Fructan, a fructose polymer with a terminal sucrose molecule, is produced by many microorganisms, and also by about 15% of plant species^1–5^. In plants, fructans are produced during vegetative development and the early stages of reproductive development, which is essential for drought and cold stress tolerance and for grain filling and yield^1–5^. Some grass cultivars accumulate high levels of fructans in mature seeds that can be used in a wide range of food and non-food application, e.g., as a functional low-calorie food. Fructan is synthesized from sucrose in the vacuole of plant cells^5^. In cereals, the trisaccharide 1-kestotriose is formed by an enzyme named Suc:Suc-1-fructosyltransferase (1-SST, EC 2.4.1.99), which transfers a fructosyl moiety from one sucrose to the fructose residue of another sucrose. An enzyme called 6G-fructosyl transferase (6G-FFT, EC 2.4.1.243) can use 1-kestotriose as a donor to transfer the fructosyl moiety to the glucose moiety of sucrose and form neokestose. The trisaccharide 6-kestotriose is formed by a key enzyme named Suc:fructan-6-fructosyltransferase (6-SFT, EC 2.4.1.10) from two sucrose molecules. Further addition of fructosyl moieties to elongate fructosyl chains of fructan from the three trisaccharides as acceptors is catalyzed by the key enzyme 6-SFT, and also by an enzyme called fructan:fructan 1-fructosyltransferase (1-FFT, EC 2.4.1.100) to form the branched graminan^2,3,6,7^. Fructan synthesis is also regulated by a transcription factor called SUSIBA1^8^. Presence of SUSIBA1 inhibits the expression of *6-SFT* and blocks fructan synthesis, high level of sucrose could inhibit *SUSIBA1* expression and then release 6-SFT to synthase fructan^8^.

The mechanism of fructan synthesis has been well understood^6–8^, but until now, it is still difficult to increase reserved fructan content in cereal grain. The function of fructan as a rapidly available energy-supplying resource during plant growth and development and tolerance and resilience to biotic and abiotic stress and environmental conditions^1–4,9,10^ mean that fructans need to be quickly hydrolyzed to meet these needs. Hydrolysis of fructan in plants is performed by fructan exohydrolases (FEHs) from a terminal of a fructose polymer in the fructan^11^. Wheat, and most probably also barley, harbor a set of different FEHs including 1-FEH (fructan 1-exohydrolase, EC 3.3.2.153)^11^ 6-FEH (fructan 6-exohydrolase, EC 3.2.1.154)^12^, 6&1-FEH^13,14^ and 6-kestotriose exohydrolase (6-KEH)^15^. Together, they hydrolyze fructan to sucrose and fructose.

Fructan is generally accepted as a dietary fiber and healthy food ingredient to promote propagation of beneficial bacteria in the digestive systems of mammals and poultry^4,16,17^. Consequently, grain fructan has gained high interest in recent years as a functional prebiotic and low-calorie healthy food and feed ingredient^2,4,17–19^. Except fructan, (1,3;1,4)-β-glucan content in barley grain is also associated with stress tolerance^20,21^ and both fructan and (1,3;1,4)-beta-glucan content are important in determining barley end uses^2,4,17–19^. Thus, development of high-fiber cereals is important for both increasing stress tolerance and healthy food production.

As a traditional method, the goal of a cross in inbred breeding is to combine traits presenting in each parent and in some case, achieve transgressive segregation^22,23^. Thus, progenies have the possibility to inherit advantage properties from their parents through segregation created by hybridization. The aims of this study were to understand the mechanism in determining final grain fructan content and to achieve high grain fructan barley with cross breeding strategy by the combination of high fructan synthesis activity and low fructan hydrolysis activity.

## Results

### A crossing strategy to achieve high grain fructan barley

**Figure 1:**
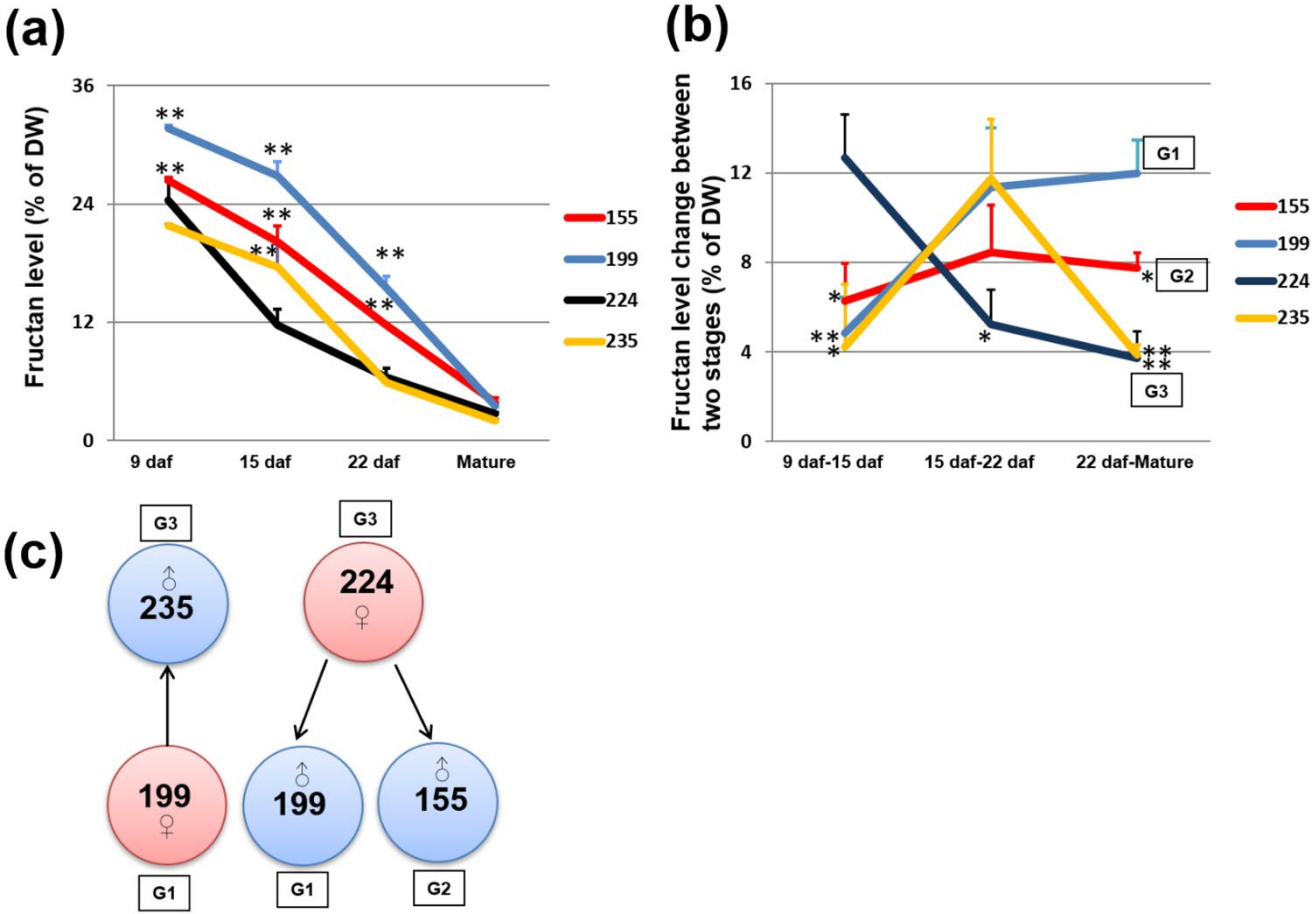
Design of the crossing strategy. (**a**) Fructan percentage per unit dry weight (DW) during grain development at 9, 15, 22 days after flowering (daf) and at grain maturity for 4 among the 12 barley lines (see Supplementary Table S1 and Supplementary Fig. S1). (**b**) Percentage change in fructan level between two development stages. Groups 1 (G1), 2 (G2) and 3 (G 3) are indicated by boxes. (**c**) Details of the crossing strategy. Pink color indicates maternal and blue color indicates paternal, arrows indicate two crossed varieties. Student’s t-test was used (Error bars show SD). *P < 0.05 and **P < 0.01 are shown for significant differences between the sample level and the lowest sample level at different stages in (**a**). *P < 0.05 and **P < 0.01 in (**b**) are shown for significant differences between the fructan reduction level of two stages and the highest reduction level. Three biological replicates or grains from three independent plants (*n* = 3) were used for analyses.

In a list of 20 barley lines^17^, based on the fructan content in mature grain, we selected 12 lines with high, medium, and low levels of fructan content. 249/Gustav, as a commercial variety, is included as a reference (Supplementary Table S1; Supplementary Fig. S1). During cultivation, we observed that lines 155, 199 accumulated relatively high fructan levels, of over 25% of dry weight (DW), at an early development stage (9 days after flowering, daf), whereas lines 224 and 235 produced relatively low fructan content at the same stage (Fig. 1a and Supplementary Fig. S1a). During grain development, the fructan content in all lines decreased gradually to between 0.6 and 3.9% of DW in mature grain (Fig. 1a; Supplementary Fig. S1d). However, we observed that during the late stages (from 22 daf to grain maturation), the total fructan content in lines 155, and 199 declined more quickly than that in lines 224 and 235 (Fig. 1a). When a diagram was plotted using total fructan level changes between the two stages, three groups were clearly distinguished (Fig. 1b). Groups 1 and 2 (G1 and G2) showed a relatively large change between 22 daf-mature grains (an over 7% change; Fig. 1b). In contrast, Group 3 (G3) showed a small change during the later stage (around 4% change; Fig. 1b). We hypothesized that it might be possible to combine the lower reduction in total fructan change at the later stage in G3 with the higher fructan level but also higher fructan reduction at late stage in G1 and G2 by crossing, to elevate the fructan content. To test this hypothesis, we selected 199, 155, 224 and 235 to perform crossing, that is, 224 (G3) was used as maternal to do cross with 199 (G1) and 155 (G2), and 199 (G1) as maternal to do cross with 235 (G3) (Fig. 1c). During screening process, 20 plants of each crossing line, as F1 generation, were cultivated, and 50 plants of each crossing line at both F2 and F3 generations were cultivated respectively, to check the segregation and select high fructan plants.

### Achieved high grain fructan barley

**Figure 2:**
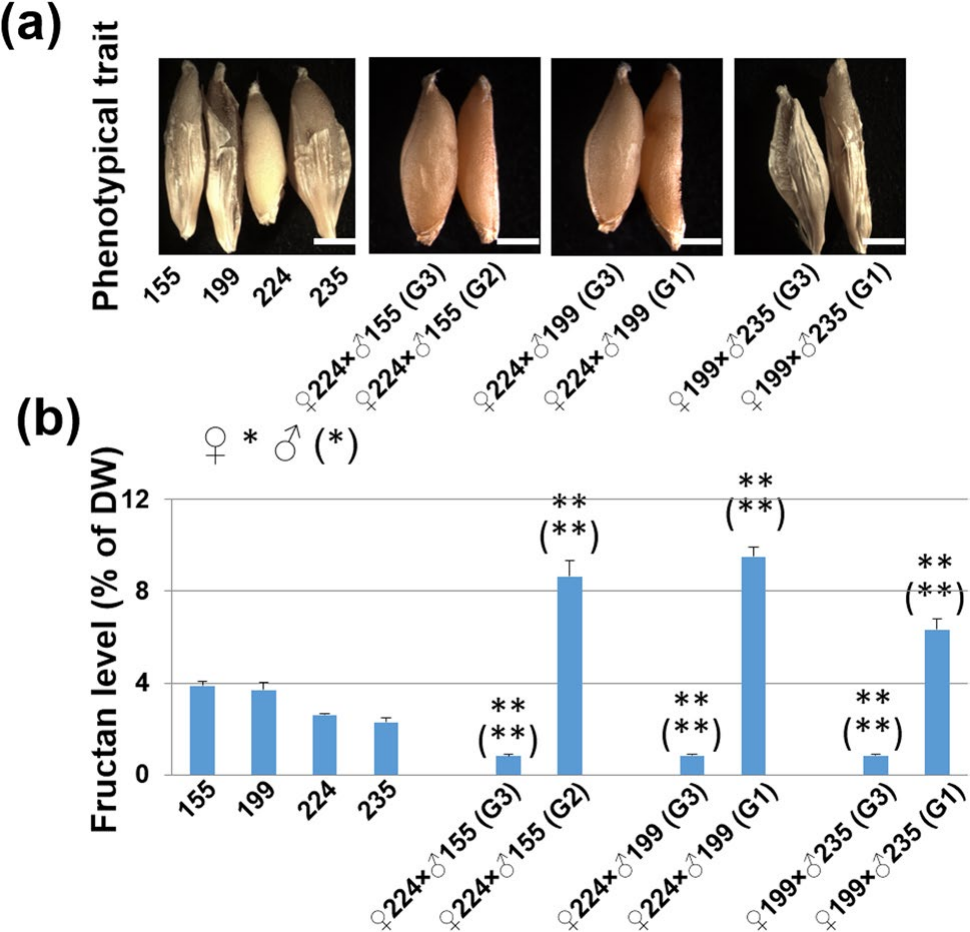
Fructan content assay in crossing lines of G1/G2 shape-inherited seed and G3 shape-inherited seed. (**a**) Images of the two types of F_3_ grains, G1/G2 shape-inherited (G1/G2) and G3 shape-inherited (G3) grains. (**b**) Percentage per unit dry weight of fructan in different kinds of grains. Student’s t-test was used (Error bars show SD). *P < 0.05 and **P < 0.01 or (*) *P* < 0.05 and (**) *P* < 0.01 in (**b**) are shown for significant differences between the progenies and the maternal or paternal line, respectively. Three biological replicates or grains from three independent plants (*n* = 3) were used for fructan analyses. Bars = 3 mm.

After segregation of the crossing progenies, two types of grain, flat and round, were generated (Fig. 2a), and from phenotypic observation, we found flat shape inherited from 199 (G1) or 155(G2), and round shape inherited from G3. When we analyzed the fructan content from ca. 100 barley grains, we found that G1 or G2 shape-inherited grains always contained much more fructan than parental [i.e. *♀*224 × *♂*155 (G2), *♀*224 × *♂*199 (G1), and *♀*199 × *♂*235 (G1)], and fructan content in G3 shape-inherited grains was very low [i.e. *♀*224 × *♂*155 (G3), *♀*224 × *♂*199 (G3), and *♀*199 × *♂*235 (G3)] (Fig. 2b). And more excitingly, we found that all the G1 or G2 shape-inherited plants also produced pure flat seeds accompanied with high fructan levels, indicating the stable hereditary property of the flat seed on morphology and fructan content. Flat seeds from F3 generation were selected as high fructan lines to continue further experiment.

### Molecular analysis interpreting high fructan mechanism

**Figure 3:**
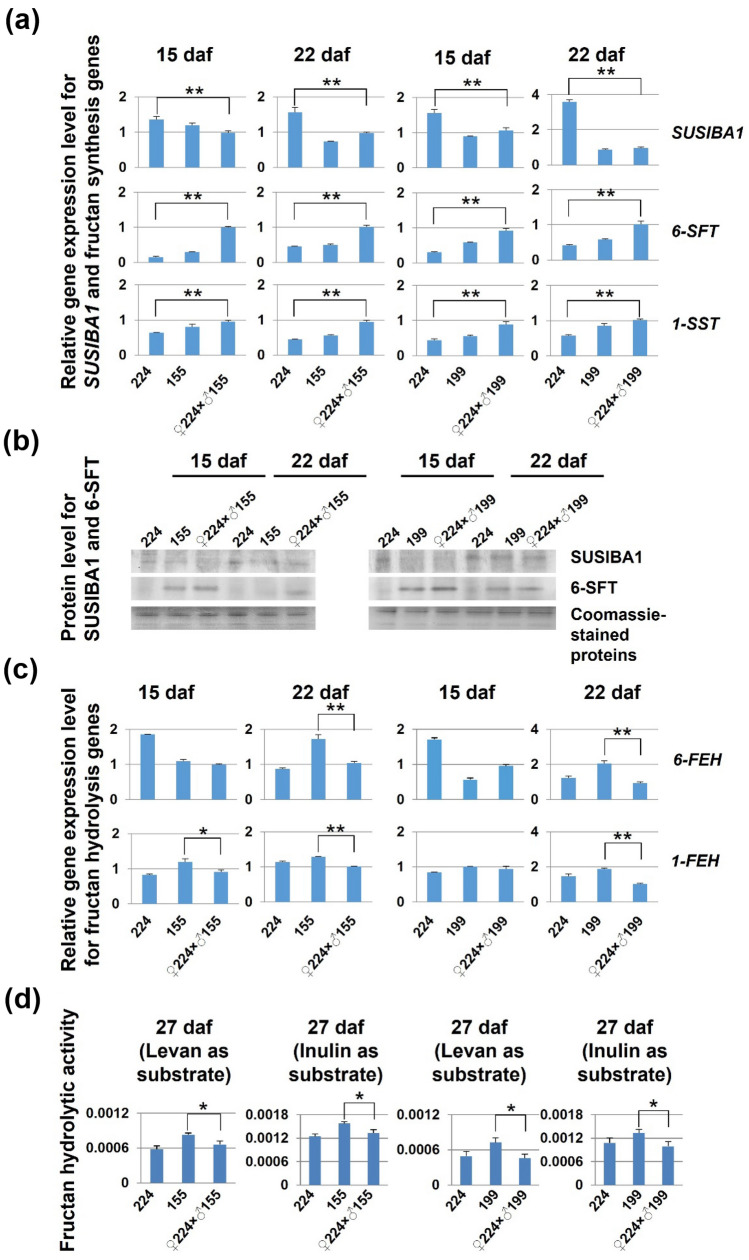
Gene and protein expression during grain development in F_3_ progenies after crossing. (**a**) Results of quantitative real-time PCR (qPCR) analysis of relative gene expression levels of a fructan negative transcription factor gene, *SUSIBA1*, and two fructan synthesis genes, *6-SFT* and *1-SST* at 15 and 22 days after flowering (daf). (**b**) Western blot analysis of proteins SUSIBA1 and 6-SFT in the corresponding samples in (**a**), and uncropped images are placed in Supplementary Fig. S2. (**c**) Results of qPCR analysis of relative gene expression levels for two representatives of fructan hydrolysis genes, *6-FEH* and *1-FEH*, at 15 and 22 daf. (**d**) Assayed enzyme activity of 6-FEH (levan as substrate) and 1-FEH (inulin as substrate) at 27 daf. Enzyme activity expressed as mg of fructose formation per min at 25 °C. Student’s t-test was used (Error bars show SD). *P < 0.05 and **P < 0.01 are shown for significant differences between the progenies and the parent of interest. Three biological replicates or grains from three independent plants (*n* = 3) were used for qPCR analysis and two biological replicates for Western blot analysis.

We use *SUSIBA1*^8^ as a negative marker for fructan synthesis and *1-FEH* and *6-FEH* as two representatives for fructan hydrolysis to follow gene expression of fructan synthesis and hydrolysis activity by qPCR during grain development*.* Two additional genes for fructan synthesis activity (*6-SFT* and *1-SST*) in validation of *SUSIBA1* regulation were also used^8^. We employed Western blot or enzyme activity assays to monitor protein products of those genes (Fig. 3 and Supplementary Fig. S2). In the developing grain of F_3_ progenies of G3 (224) × G2 (155) and G3 (224) × G1 (199), high expression of synthetic activity (i.e., low expression of the negative transcription factor SUSIBA1 and high expression of 6-SFT and 1-SST) was inherited by the progenies from the parents of G2 and G1 at both early (15 daf) and late (22 daf) stages (224) (Fig. 3a,b and Supplementary Fig. S2). Interestingly, hydrolysis activity consistently decreased at gene expression levels at the late stage (22 daf) in the progenies of G3 (224) × G2 (155) and G3 (224) × G1 (199) compared with G2 (155) and G1 (199) (Fig. 3c). When we examined the fructan hydrolysis activity for both 6-FEH (levan as substrate) and 1-FEH (inulin as substrate) (Fig. 3d), we found that it was also declined significantly at the late stage (27 daf) in the progenies of G3 (224) × G2 (155) and G3 (224) × G1 (199) (Fig. 3d). The expression pattern of high synthesis activity and low hydrolysis activity was also obtained in the progenies of G1 (199) × G3 (235)^8^.

### Fructan metabolic patterns revealing the success of the crossing strategy

**Figure 4:**
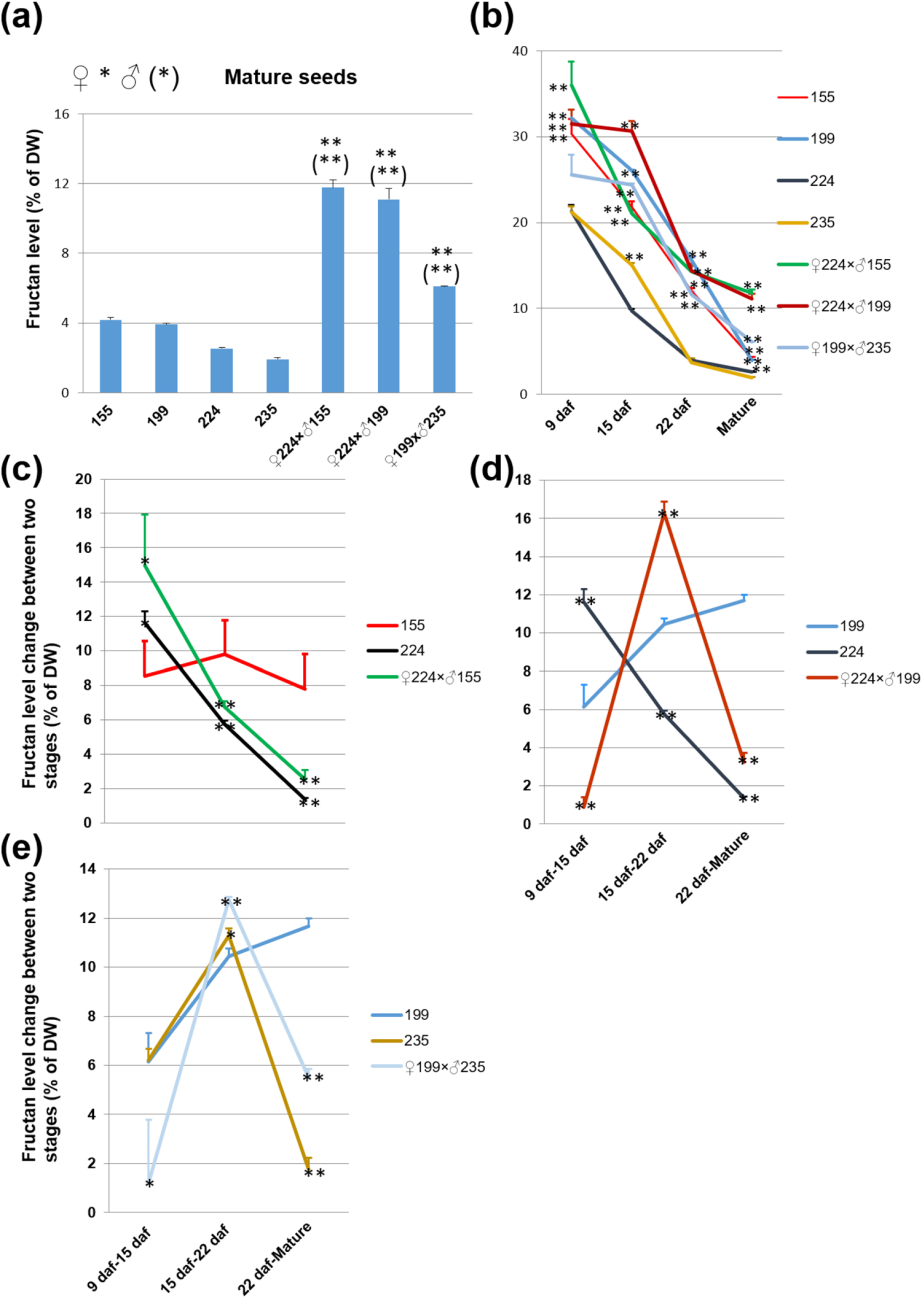
Fructan percentage per unit dry weight (DW) in the high grain fructan barley. (**a**) Fructan level in mature grains of different barley lines, F_3_ progenies of G3 (224) × G2 (155), G3 (224) × G1 (199) and G1 (199) × G3 (235), and the parents 155, 199, 224, and 235. (**b**) Fructan level during grain development in the same barley lines. (**c**–**e**) Fructan level percentage change between two development stages of individual barley lines. Student’s t-test was used (Error bars show SD). **P < 0.01 is shown for significant differences between the progenies and the parents in (**a**). **P < 0.01 is shown for significant differences between the sample level and the lowest sample level at different stages in (**b**). *P < 0.05 and **P < 0.01 are shown for significant differences between the progenies/G3 (224 or 235) and G1/G2(199/155) in (**c**–**e**). Three biological replicates or grains from three independent plants (*n* = 3) were used for analyses.

After molecular mechanism characterization, we carefully scrutinized the phenotypic traits of F_3_/F_4_ progenies with the focus on the fructan content trait (Fig. 4; Supplementary Fig. S3) and other important agronomical traits (Supplementary Fig. S4).

In mature grain of the progenies of 224 × 155, 224 × 199 and 199 × 235, the fructan content increased to 11.8%, 11.1% and 6.1% respectively, compared with the level in their parents (3.9% in 199, 4.2% in 155, 2.6% in 224 and 1.9% in 235) (Fig. 4a; Supplementary Fig. S3d). When we checked the fructan content during grain development, we observed that the progeny lines of 224 × 155, 224 × 199 and 199 × 235 followed their parents of 155 and 199, but not 224 and 235, at the earlier stages and accumulated high fructan content, around 12% before 22 daf (Fig. 4b; Supplementary Fig. S3a–c). After 22 daf, the fructan content in the progenies of 224 × 155, 224 × 199 and 199 × 235 decreased more slowly than in their parents G2 and G1 and followed the G3 pattern. When we plotted the reduction changes between two stages (Fig. 4c–e), the results showed that the progenies of G3 (224) × G2 (155), G3 (224) × G1 (199), and G1 (199) × G3 (235) followed the pattern of G3 particularly between 22 daf-mature stages with a less than 6% change, and did not follow G1 and G2 with a significant difference (Fig. 4c–e). Thus, we concluded that the high grain fructan content in lines of G3 (224) × G2 (155), G3 (224) × G1 (199) and G1 (199) × G3 (235) was due to a combination of high synthetic activity of G2 and G1 and low hydrolytic activity of G3.

After characterization of the fructan content trait in the obtained barley lines in phytotron, we examined other agronomical traits to see whether these are affected by the trait of high grain fructan content. It was found that all six traits examined in the progenies were similar or intermediate to those in the paternal and maternal material, and that there was no marked impact on the traits due to the increase in grain fructan (Supplementary Fig. S4). For example, one of the most important traits, thousand kernel weight, was higher in the lines of G3 (224) × G2 (155) and G3 (224) × G1 (199) than in their paternal lines 155 and 199, but lower than in their maternal 224 (Supplementary Fig. S4f).

### Correlation between fructan and beta-glucan content in high-fructan barley grain

**Figure 5:**
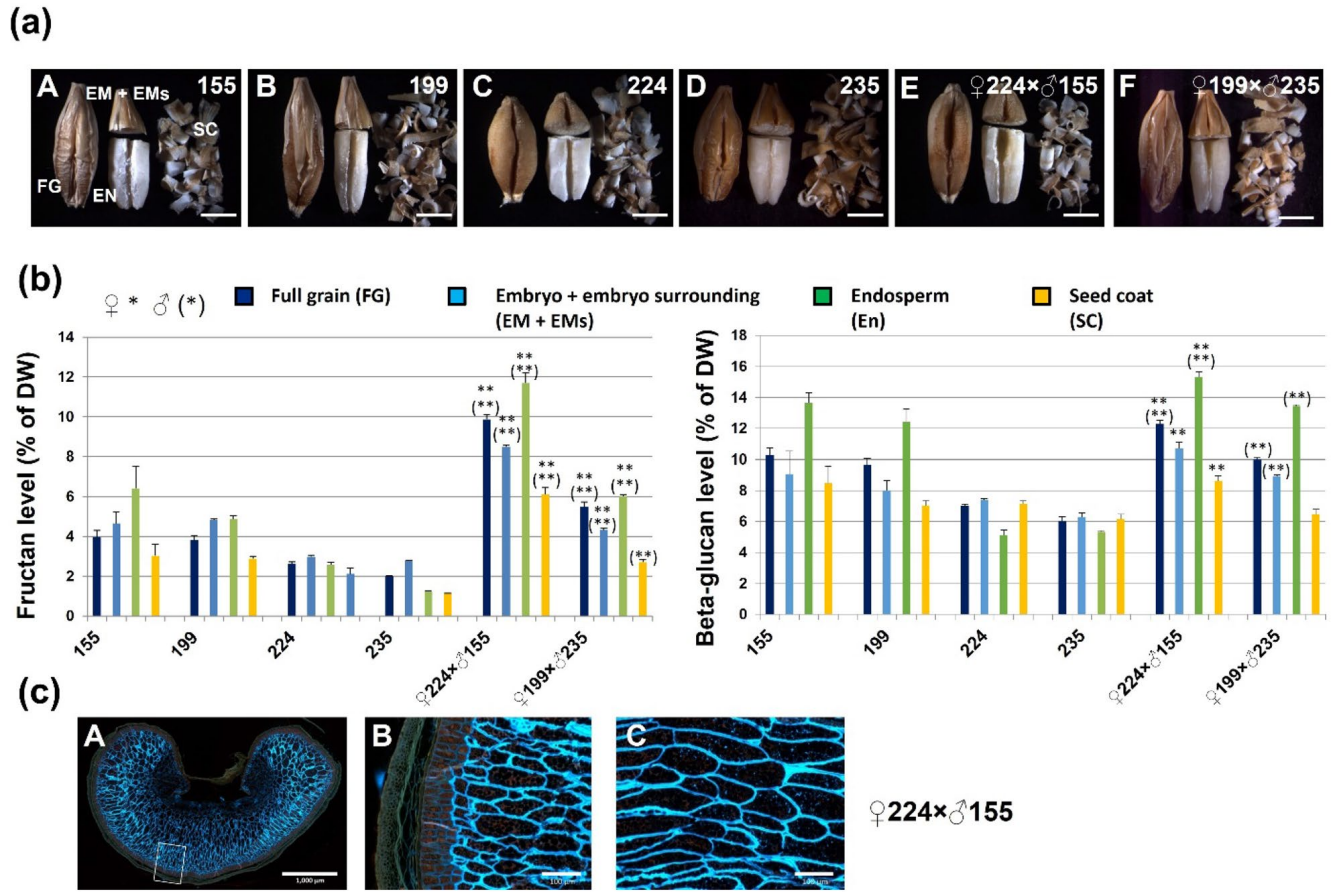
Fructan and beta-glucan levels and microstructure of different tissues in high fructan grain. (**a**) Images of the front side (A–F) of a full grain (FG, dehusked), embryo (EM), embryo-surrounding tissue (EM + Ems), endosperm (EN), and seed coat (SC). Tissues of barley lines 155, 199, 224, 235, 224 × 155 and 199 × 235 were hand-dissected using a razor blade. (**b**) Percentage of fructan and beta-glucan per unit dry weight (DW) in the different tissue fractions of the barley lines. A good correlation between fructan and beta-glucan content was found in full grain and all three tissues (Pearson correlation coefficient (*r*) = 0.9121). (**c**) Microscopic images of the microstructure in high-fructan grain line 224 × 155. Photographs of cross-sections of full grains (A). Close-ups of the corresponding boxed region (B, C) in the images (A), respectively. Student’s t-test was used (Error bars show SD). *P < 0.05 and **P < 0.01 or (*) *P* < 0.05 and (**) *P* < 0.01 in (**b**) are shown for significant differences between the progenies and the maternal or paternal line, respectively. Three biological replicates or grains from three independent plants (*n* = 3) were used for analyses. Bars = 3 mm (A–F of **a**), 1000 µm (A of **c**), 200 µm (B of **c**), and 100 µm (C of **c**).

In order to check the correlation between fructan and beta-glucan, we investigated fructan and beta-glucan content in different lines of F_3_/F_4_ progenies. Interestingly, we observed a good correlation between fructan and (1,3;1,4)-beta-glucan content between different lines with Pearson correlation coefficient (*r*) of 0.87 (Supplementary Fig. S5). When we hand dissected full grain (FG, dehusked grain) into three fractions, germ (embryo and embryo surrounding or Em + EMs), endosperm (En), and seed coat (SC) (Fig. 5a), interestingly, we found that the endosperm tissue of G1, G2 and high fructan level crossing lines had highest fructan and beta-glucan content among the three fractions analyzed, which is different with G3 (Fig. 5b). A good correlation between fructan and beta-glucan content in the full grain and all three tissues of different lines was also found, with Pearson correlation coefficient (*r*) of 0.91 for fructan and beta-glucan content. To identify the locations of beta-glucan in the endosperm tissue, we subjected the line of high grain fructan barley 224 × 155 to microscopic analysis. The results showed that the stained beta-glucan intensity in cell walls of aleurone, subaleurone, (Fig. 5c). Taken together, the endosperm tissue of high grain fructan barley produced a high content of fructan and beta-glucan, indicating a good correlation between fructan and beta-glucan, and good potential for increasing seed stress tolerance and improving healthy food production with our crossing strategy.

### Fructan content and yield of the achieved barley under field conditions

Since fructan is an easily mobilized carbohydrate^2–4^, particularly in stress conditions, an important question is whether the high grain fructan barley can also produce high fructan content in grain under field conditions. From the field trial in 2018, we found that under field conditions, the high grain fructan barley lines (224 × 155, 224 × 199 and 199 × 235) of F4 generation produced significantly higher fructan content in mature grain than their parents (155, 199, 224 and 235) (Supplementary Fig. S6). Other important agricultural traits of the high grain fructan barley examined in the field trial were found to be the same as, or intermediate between, the paternal or maternal material (Supplementary Fig. S7). There was no obvious impact on the traits at current generation due to the increase in grain fructan (Supplementary Fig. S7).

**Figure 6:**
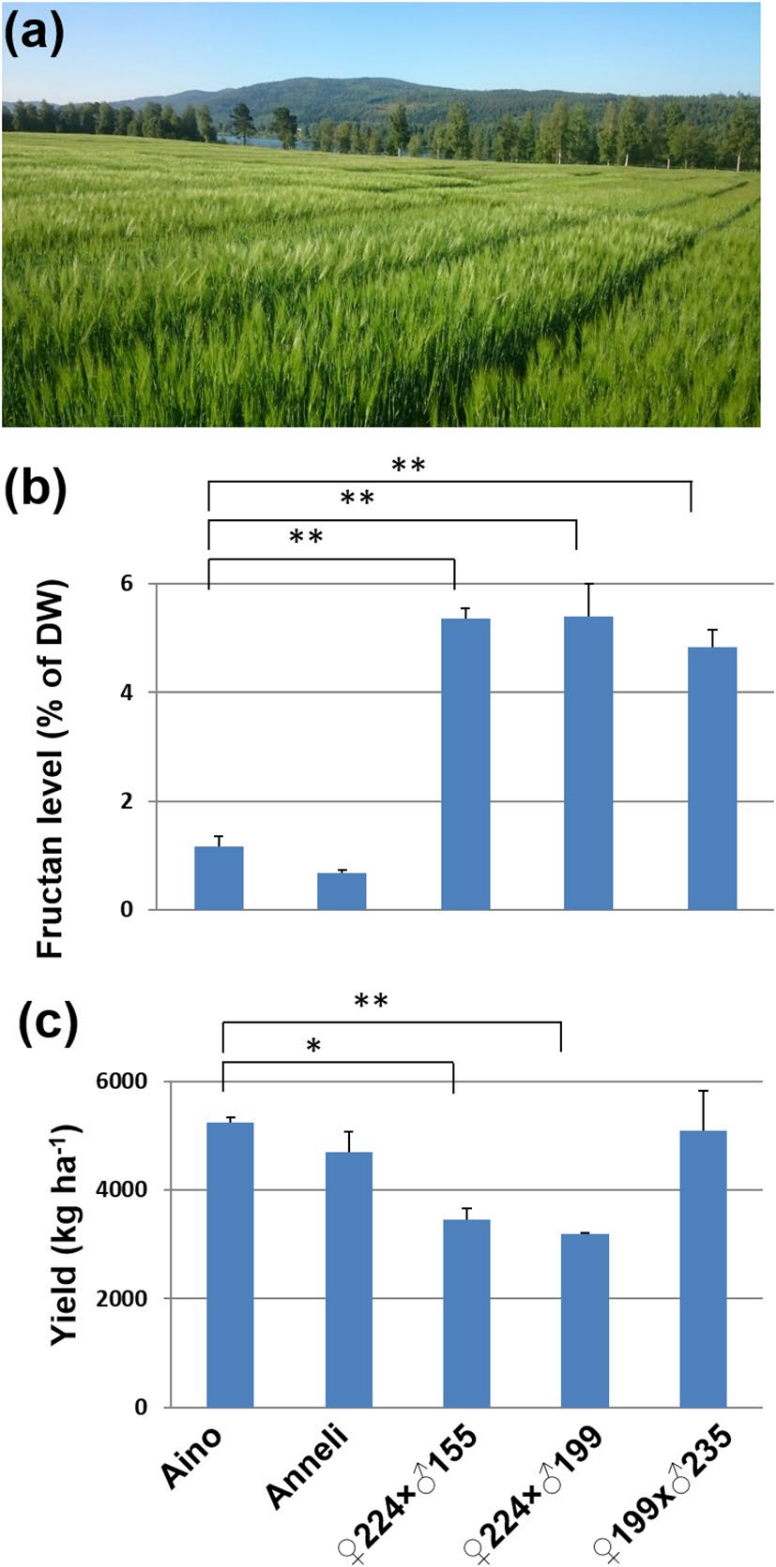
A large field trial to assess fructan levels and yield. (**a**) Photo of a large field trial. (**b**) Fructan content in the field trial samples. (**c**) Yield of two elite varieties, Aino (6 row) and Anneli (2 row), and F_5_ crosslines of high grain fructan barley. Student’s t-test was used (Error bars show SD). *P < 0.05 and **P < 0.01 are shown for significant differences between high-fructan barley and elite varieties. Plot of 15 m^2^ for each line and two plots were performed following the agricultural company´s test system. Three biological replicates or grains from three independent plants (*n* = 3) were used for fructan analyses.

In 2019, the three barley crosslines of F5 generation were applied to a large field trial (Fig. 6a). All lines showed significantly higher fructan content, 2.5–4.6 fold higher than that in Aino and 4.3–7.9 fold higher than that in Anneli (Fig. 6b). The yield assessment showed that, compared with elite variety, Aino, the lines of 244 × 155 and 224 × 199 had lower yield (Fig. 6c). Excitingly, the barley line of 199 × 235 has similar yield compared with Aino at F5 generation and produces ~ 4.1 fold higher fructan (Fig. 6b,c).

### Starch and small sugar analysis in the high grain fructan barley

When examined starch granules in the barley by staining with an iodine solution, we observed that lines 224, 235 and 224 × 199 are typical waxy mutants with a reddish color. 249/Gustav, as a commercial variety, is included as a reference (Supplementary Fig. S8). Electron microscopy confirmed that they are waxy mutants with many small granules (B-type granules) and slightly enlarged big granules (A-type granules) (Supplementary Fig. S9). Analysis of starch and amylose content indicated that starch content in all lines is over 44.7% per unit dry weight (Supplementary Fig. S10a), and amylose content in four lines, 224, 235, 224 × 155 and 224 × 199, is significantly lower than the commercial variety 249/Gustav (Supplementary Fig. S10b). Lines 224 and 224 × 199 are typical waxy mutants and 235 and 224 × 155 are near waxy mutants according to Asare et al.^24^. Other lines are normal or similar to the control 249/Gustav with over 32% of amylose (Supplementary Fig. S10b). We further analyzed proteins associated with starch granules (Granule Bound Starch Synthase I: GBSSI) and found that the low amylose content correlates with less GBSSI protein amount from starch granules compared with Gustav, which confirms that the lines of 224, 235, 224 × 155 and 224 × 199 are waxy mutants or near waxy mutants (Supplementary Fig. S10c). Small sugar analysis indicated that all waxy or near waxy mutant lines (224, 235, 224 × 155 and 224 × 199) have relatively higher small sugar content than Gustav, as opposed to the crosslines of 199 × 235 (Supplementary Fig. S10d). So, we can conclude that the mechanism in the high fructan line of 199 × 235 is different with other crossing lines, which has more potential for future applying.

## Discussion

We have achieved high grain fructan barley by using genetic variations in fructan synthesis and in fructan hydrolysis activity via crossbreeding, which can be used for agricultural production of high dietary fiber grain. In this study, we successfully introduced low fructan hydrolysis activity into high fructan level varieties, for fructan accumulation (Fig. 3c,d). This indicates an important role of fructan hydrolysis activity in high grain fructan breeding for cereals. A concern with the barley exhibiting low fructan hydrolysis activity is that the low activity may affect fructan remobilization from stems for stress tolerance and grain filling. In our field trials, we did not observe such a problem. When we measured the fructan content in the stems of penultimate internode before and after flowering in field trial samples, we found all lines to remobilize stem fructan completely when seeds are mature (Supplementary Fig. S11).

During the process of high fructan lines screening, we found that all the G1 or G2 shape-inherited seeds could produce progenies with unified flat grains accompanied with high yield of fructan [i.e.* ♀*224 × *♂*155 (G2), *♀*224 × *♂*199 (G1), and *♀*199 × *♂*235 (G1)] (Fig. 4). Here, we conclude that high fructan property in flat seed can be stably inherited into next generation without morphology segregation. As a screening marker, the phenotype of flat seed increases the speed of high fructan lines screening. Meanwhile, we cannot exclude that the genotyping inside the flat lines is pure because of the complexity of gene recombination during hybridization process.

**Figure 7:**
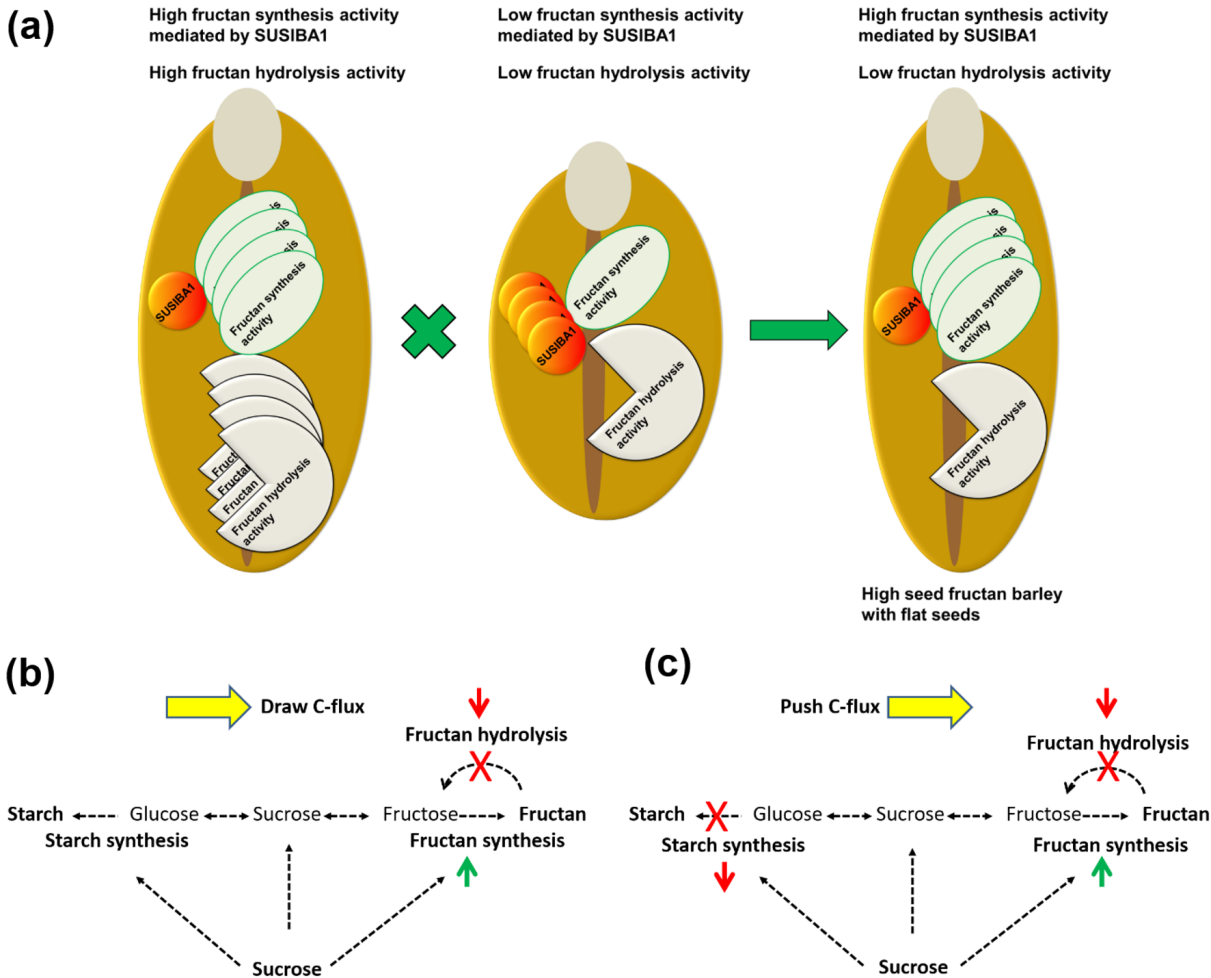
Model depicting the crossing strategy in this study to obtain high grain fructan barley and mechanism in determining grain fructan content at harvesting. (**a**) The red disc represents negative transcription factor SUSIBA1 for fructan synthesis. Fructan synthesis activity as indicated represents at least activity of 6-SFT and 1-SST. Fructan hydrolysis activity consists at least of 6-FEH and 1-FEH. Less SUSIBA1 leads to more fructan synthesis activity. High grain fructan barley usually has a flat grain phenotype. (**b**) Lower fructan hydrolysis activity (pull marker) together with the higher fructan synthesis activity (push maker) generates a force for carbon-flux to fructan accumulation without impacting starch synthesis. (**c**) Starch synthesis is disrupted by mutations, which generates a high concentration of small sugars pushing carbo-flux to fructan synthesis accompanied with lower fructan hydrolysis and higher fructan synthesis activity.

Cereal grain with a high ratio of fructan/beta-glucan to starch/small sugars would have a relatively low calorie value and could be used as a preferred ingredient in healthy food products^2^. However, higher fructan content can lead to lower starch content in barley grain (Supplementary Fig. S4), which can potentially impact yield^25,26^. In this study, we devised a crossing strategy through binding early-stage high fructan synthetic activity with late-stage low fructan hydrolytic activity to generate high grain fructan barley. The crossing strategy is summarized as a model in Fig. 7a. In the model, fructan hydrolysis activity comprises the activity of 1-FEH, 6-FEH and other FEHs. Based on the model, we suggest that combining a variety with low expression of *SUSIBA1* at an early stage of grain development with a variety with low hydrolytic activity at a late stage is important for achieving high fructan content in cereal grain. In the model (barley line 199 × 235), we have increased fructan synthesis and decreased fructan hydrolysis, which pulls carbon-flux to fructan accumulation, starch synthesis and yield are not affected. (Fig. 7b). When starch synthesis is disrupted by mutations, higher concentration of small sugars pushes carbon-flux to fructan synthesis, and the yield was impacted, and meanwhile, decreased hydrolysis activity pulls carbon-flux to fructan, i.e. 224 × 155 and 224 × 199 (Fig. 7c). The co-existing of “push” and “pull” mechanisms leading to an extremely high fructan content (near 12%). Thus, we recommend that to achieve high grain fructan barley without a yield penalty, a starch synthesis disruption should be avoided or excluded via segregation, e.g. line 199 × 235 (Fig. 6). However, if high fructan content is the only factor for commercial purpose, the breeding method with the co-existing of “push” and “pull” is suggested.

## Materials and methods

### Source of barley materials, growth conditions, barley crossing and field trails

Barley varieties 120 and 131 were donated by NordGen, Sweden; 181, 198 and 199 were contributed by The Royal Veterinary and Agricultural University, Denmark; 224, 235 and 236 came from our collaborator, Lantmännen Sweden; 220, 228 and 249 were stocked in our group. Barley plants were cultivated in phytotrons, with conditions of 9 h light (300 µmol m^−2^ s^−1^) at 12 °C, 15 h darkness at 8 °C, 60% relative humidity for the first 4 weeks, and then to 16 h light (400 µmol m^−2^ s^−1^) at 20 °C, 8 h darkness at 12 °C, and 70% relative humidity until maturation. The Zadoks scale^27^ was used to specify developmental stages. Labeled spikes were harvested at 9, 15, 22, and 27 days after flowering (daf) and at full maturity. Five grains from middle positions of each row in each spike were used. Stems were sampled from plants at 3:00 pm in a field trial and put immediately into liquid nitrogen and kept at − 80 °C until use. Stems of penultimate internode were used for fructan analysis.

Barley crossing was performed according to Poehlman and Sleper^28^. and the detail crossing strategy was described in Fig. 1. Briefly, 224 (G3) was used as maternal to do cross with 199 (G1) and 155 (G2), and 199 (G1) as maternal to do cross with 235 (G3). After crossing, 20 plants of each crossing line, as F1 generation, were cultivated, and for F2 and F3 generations, 50 plants of each crossing line were cultivated respectively, to check the segregation and select high fructan plants. Based on the results we found in this paper, flat seed is correlated with high fructan. After three-generation screening, the F_3_ progenies of barley lines with unique flat seeds were defined with high fructan lines and used for further experiments. The F_4_ grain was used for a small field trial and the F_5_ grain was used for yield assessment.

Field trials were performed in 2018 and 2019, respectively. In 2018, area with 20 m^2^ was separated into four independent field blocks of around 4 m^2^ per block, at a site (59.8222° N, 17.6593° E) near Uppsala, Sweden. Three replicates of three barley lines of F4 generation (224 × 155, 224 × 199, 199 × 235) and their parents (155, 199, 224, 235) were randomly distributed in the four blocks. Harvested grains were stored dry at room temperature for different experiments. In 2019, A large field trial was performed by Lantmännen at Bjertorp, Sweden, 355 km from Uppsala, to test yield. Trial seeds (F_5_) were multiplied in New Zealand. Two plots of 15 m^2^ per plot size were used for three cross lines (224 × 155, 224 × 199, 199 × 235). Two elite barley varieties, one six-row barley Aino approved in Norway and one two-row barley Anneli on the Swedish market were included in the yield assessment.

### Primers

Primers were listed in Supplementary Table S2.

### Determination of fructan, beta-glucan, amylose and starch and small sugars

Seeds or stems were sampled from 3:00 pm and grinded in liquid nitrogen to powders. Fructan, beta-glucan, amylose and starch were isolated and analyzed as described previously (Nemeth et al.^17^ for fructan; Sun et al.^29^ for starch and amylose; Zhang et al.^30^ for beta-glucan), and according to protocols provided in the kits from Megazyme (Bray, Co. Wicklow, Ireland) with kit number K-FRUC for fructan, K-BGLU for beta-glucan, K-AMYL for amylose and K-TSTA for total starch. d-fructose, d-glucose/sucrose and maltose were analyzed according to protocols provided in the kits from Megazyme with kit number K-FRUGL for d-fructose, K-SUCGL for d-glucose/sucrose and K-MASUG for maltose. Hydrolysis of fructan was calculated by subtraction of fructan content at an earlier stage with fructan content at a later stage.

### qPCR analysis

All the samples were harvested from 3:00 pm and ground to a fine powder in liquid nitrogen. Total RNA was isolated using Spectrum Plant Total RNA kit (Sigma-Aldrich, USA) according to the manufacturer’s instructions. Total RNA (1 μg) was used to synthesize cDNA with the first Strand cDNA Synthesis Kit (Quanta Biosciences, USA). 15 ng cDNA was used for qPCR analysis. qPCR was completed in a 20 μL solution containing 5 μM specific primers and 2 × SYBR Green/Fluorescein qPCR Master Mix (thermo scientific), and program was as follows: 95 °C for 4 min; 40 cycles of 95 °C for 10 s and 60 °C for 30 s. The melting curve analysis was completed by increasing the temperature from 60 to 95 °C at a rate of 0.05 °C s^−1^. Relative expression levels of genes related with fructan metabolism in this manuscript were calculated using the comparative *C*t method^8,31^. Gene expression levels normalized against the values of the housekeeping gene *Ubiquitin10*. All the primers used for qPCR were listed in Supplementary Table 2.

### Granule-associated protein isolation and analysis

The granule-associated protein was isolated according to the method described before^32^ with slight modifications. Briefly, seeds of 15 daf spikes were sampled at 3:00 pm and grinded in liquid nitrogen. Powders of 300 mg was homogenized in 2 ml starch extraction buffer at 4 °C. After filtration and centrifugation, the starch pellet was washed with starch extraction buffer, cold 95% ethanol and acetone as described. The granule protein was released by boiling in 500 µl gelatinization buffer. Granule proteins were precipitated after mixing with cold acetone. The air dried pellet was solubilized in 50 µl soluble buffer. The isolated proteins were analyzed on SDS-PAGE and stained with Coomassie.

### Western blot analysis

All the materials were sampled from 3:00 pm and ground into fine powder with liquid nitrogen. 100 mg powder was used for total protein isolation with Protein Extraction Reagent Type 4 (Sigma-Aldrich, USA). Briefly, 1.2 ml of cold methanol (4 µl protease inhibitor cocktail/ml methanol) was added into 100 mg powder and then proceed followed the manual. Isolated total protein was loaded in NuPAGE 4–12% Bis–Tris Gel (Invitrogen, Carlsbad, CA, USA) and then stained with Coomassie blue after separation with MOPS SDS Running Buffer (Invitrogen, Carlsbad, CA, USA). Protein levels were normalized based on the staining signal. Separated proteins were transformed into PVDF membrane with transfer buffer (Invitrogen, Carlsbad, CA, USA) and the blot of primary and second antibodies was followed overnight and for 2 h respectively after washing with TBS and TBST solution. Membrane staining was performed with 1-Step NBT/BCIP (Thermo Scientific, USA). The primary antibodies specific to SUSIBA1 and 6-SFT were used^8^ with the same blot patterns as previous publication^8^. The blot sizes of SUSIBA1 and 6-SFT are 30 kDa and 23 kDa respectively, and High-Range protein marker (Merk, Cat. GERPN756E) was used. Cropped gels and blots are displayed in Fig. 3b in order to improve the clarity and conciseness of presentation. Uncropped images are placed in Supplementary Fig. S2.

### Light microscopy and scanning electron microscopy (SEM)

Microscopy to study the microstructure of barley grains was performed by a commercial service at VTT Technical Research Centre of Finland Ltd. Only highest fructan content barley lines (grain of 224 × 155) was selected due to high costs. Barley grains were cut into halves by cross-section. The halves were fixed in 1% (v/v) glutaraldehyde in 0.1 M Na–K phosphate buffer (pH 7.0), dehydrated in an ethanol series, and embedded in hydroxyethyl methylacrylate resin. Polymerized samples were sectioned in a rotary microtome HM 355S (Microm Laborgeräte GmbH, Walldorf, Germany). The sections were transferred onto glass slides and stained. For beta-glucan observation, the slides were stained with aqueous 0.01% (w/v) Calcofluor White (Fluorescent brightener 28, Aldrich, Germany)^33,34^. The stained cross-sections were examined under light excitation (390–420 nm; emission > 450 nm) by epifluorescence microscopy with a Zeiss AxioImager M.2 microscope (Carl Zeiss GmbH, Göttingen, Germany). Micrographs were obtained using a Zeiss Axiocam 506 CCD color camera.

Starch was randomly scratched from a central endosperm region to a slide and stained with drops of an iodine solution^35^. The starch/iodine solution were covered by coverslips and observed on a light microscope of Zeiss Axioscope A1 with 200 magnification. For SEM, a cross-section of a central endosperm region was observed on a SEM–EDS Hitachi TM-1000-µ-Dex tabletop scanning electron microscope with 2000 magnification as described^36^.

### Enzyme activity assay

The activity of fructan hydrolases was assessed as described previously^13^ with minor modifications. In brief, developing grain harvested at 27 daf was grinded and 1 ml of extraction buffer was added to 0.3 g of powder, homogenized, and centrifuged. Small sugars in the supernatant were removed by microspinG-50 columns (GE Healthcare Bio-Sciences, Uppsala, Sweden). To measure the activity of 1-FEH and 6-FEH, aliquots of the supernatant were incubated with 3% (w/v) commercial chicory inulin and 3% (w/v) levan for 1 h at 25 °C, and stopped by heating. After centrifugation, the supernatant was used to measure released fructose according to protocols provided in the kits from Megazyme. Enzyme activity was expressed as mg of fructose formation per min at 25 °C.

### Statistics

Statistical significance between different groups was analyzed using 2-sided unpaired Student’s t-test. Results are expressed as mean ± SD. P values < 0.05 were considered as significant: * < 0.05, ** < 0.01.

Experimental research and field studies on plants were in compliance with relevant institutional, national, and international guidelines and legislation.

### Source clarification of barley varieties

The authors clarify that barley varieties used in this paper came from NordGen, Sweden, The Royal Veterinary and Agricultural University, Denmark, Lantmännen Sweden and our group, respectively, and all the varieties are permitted for research using purpose.

## Supplementary Information

Supplementary Information 1.

Supplementary Information 2.

## Data Availability

The data generated and analysed during the current study are available in the Source data.
