# Supplementary material for: Achieving of high-diet-fiber barley via managing fructan hydrolysis
Source: Sci Rep. 2022 Nov 9;12:19151. doi: 10.1038/s41598-022-21955-1 (PMC9646770; doi:10.1038/s41598-022-21955-1)
Supplement: Supplementary file 2 — Supplementary Information 2. [file 41598_2022_21955_MOESM2_ESM.docx]

**Achieving of high-diet-fiber barley via managing fructan hydrolysis**

*Mingliang Fei^1,2,3†^, Yunkai Jin^2†^, Jia Hu^2^, Gleb Dotsenko^4^, Ying Ruan^1,3*^, Chunlin Liu^3,5^, Gulaim Seisenbaeva^4^, Annica AM Andersson^4^, Roger Andersson^4^ and Chuanxin Sun^2*^*

^1^Key Laboratory of Crop Epigenetic Regulation and Development in Hunan Province, Hunan Agricultural University, Changsha, 410128, China

^2^Department of Plant Biology, Uppsala BioCenter, Linnean Centre for Plant Biology, Swedish University of Agricultural Sciences, P.O. Box 7080, SE-750 07 Uppsala, Sweden

^3^Key Laboratory of Education Department of Hunan Province on Plant Genetics and Molecular Biology, College of Bioscience and Biotechnology, Hunan Agricultural University, Changsha 410128, China

^4^Department of Molecular Sciences, Uppsala BioCenter, Swedish University of Agricultural Sciences, P.O. Box 7015, SE-750 07 Uppsala, Sweden

^5^College of Agronomy, Hunan Agricultural University, Changsha, 410128, China

**^#^These authors contributed equally to this work.**

***Authors for correspondence:**

Chuanxin Sun

Department of Plant Biology, Uppsala BioCenter, Linnean Centre for Plant Biology, Swedish University of Agricultural Science (SLU), P.O. Box 7080, SE-75007 Uppsala, Sweden

Email: [Chuanxin.Sun@slu.se](mailto:Chuanxin.Sun@slu.se); Phone: +46-18-673252

**ORCID**

Chuanxin Sun, <https://orcid.org/0000-0003-2755-0443>

Ying Ruan

Key Laboratory of Crop Epigenetic Regulation and Development in Hunan Province, Hunan Agricultural University, Changsha, 410128, China; Key Laboratory of Education Department of Hunan Province on Plant Genetics and Molecular Biology, College of Bioscience and Biotechnology, Hunan Agricultural University, Changsha 410128, China

Email: [yingruan@hotmail.com](mailto:yingruan@hotmail.com); Phone: +86-13808480429

**Fei *et al.,* 2022. Supplementary information (Supplementary Figures S1-S11 and Tables S1, S2)**

**
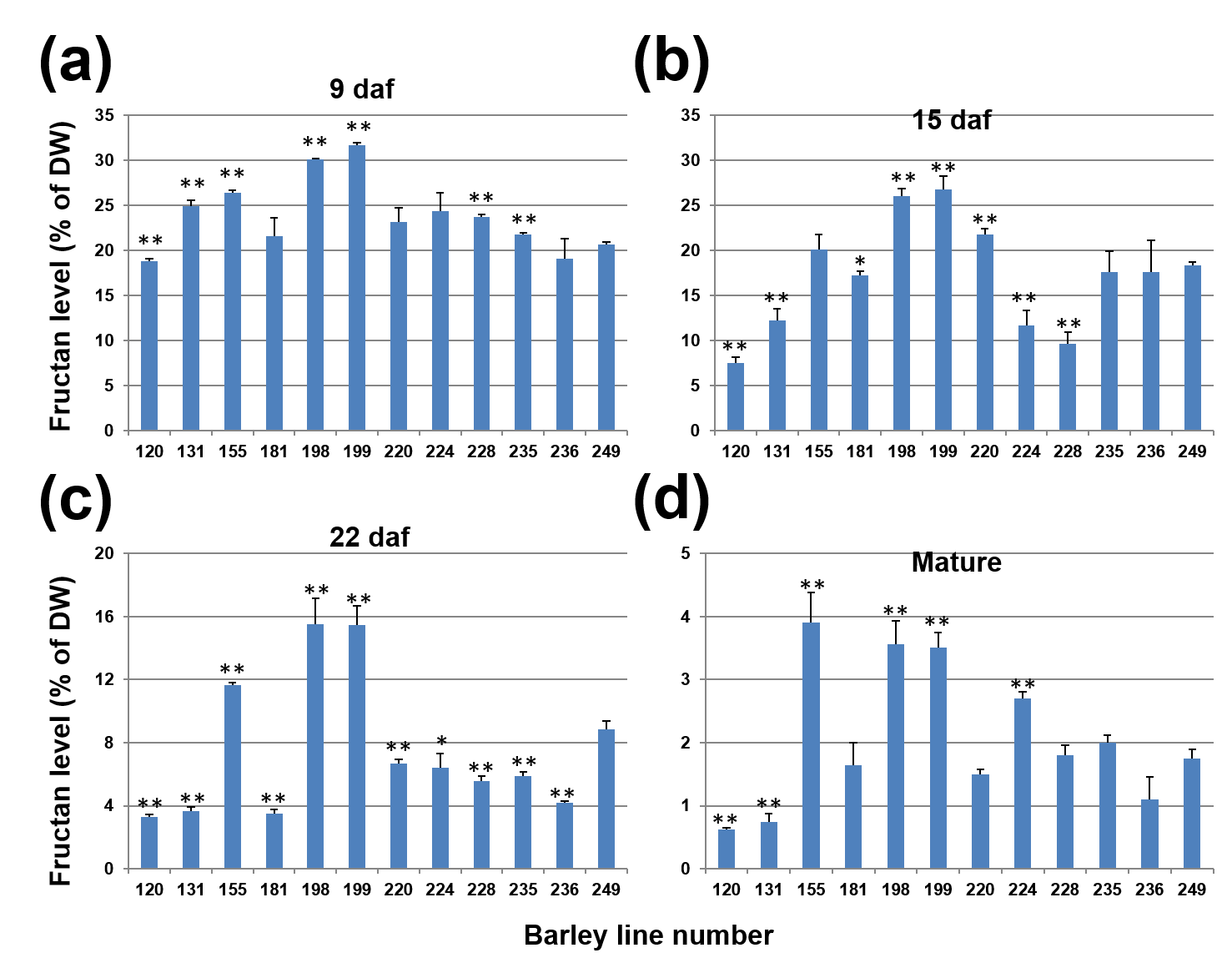
**

**Fig. S1** Fructan percentage per unit dry weight (DW) during grain development at 9 (**a**), 15 (**b**), and 22 (**c**) days after flowering (daf) and at grain maturation (**d**) for the 12 barley lines listed in Table S1. Student’s t-test was used (Error bars show SD). **P* < 0.05 and ***P* < 0.01 are shown for significant differences between barley lines and commercial variety 249 (a normal and traditional Swedish variety called Gustav) at different stages. Mid-spike grains from three independent plants (*n* = 3) were used for fructan determination.

**
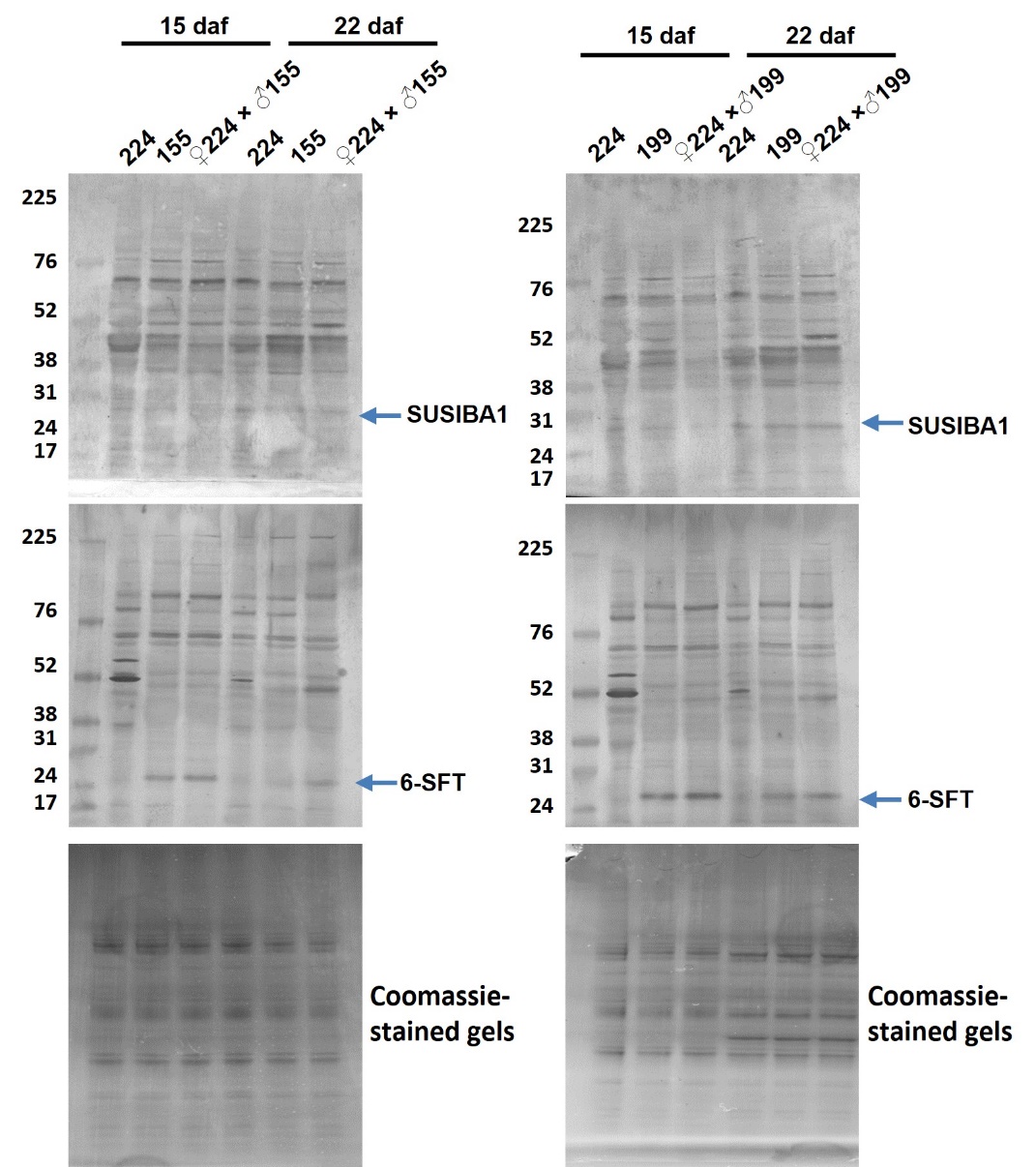
**

**Fig. S2** Uncropped gels and plots corresponding to Figure. 3b

**
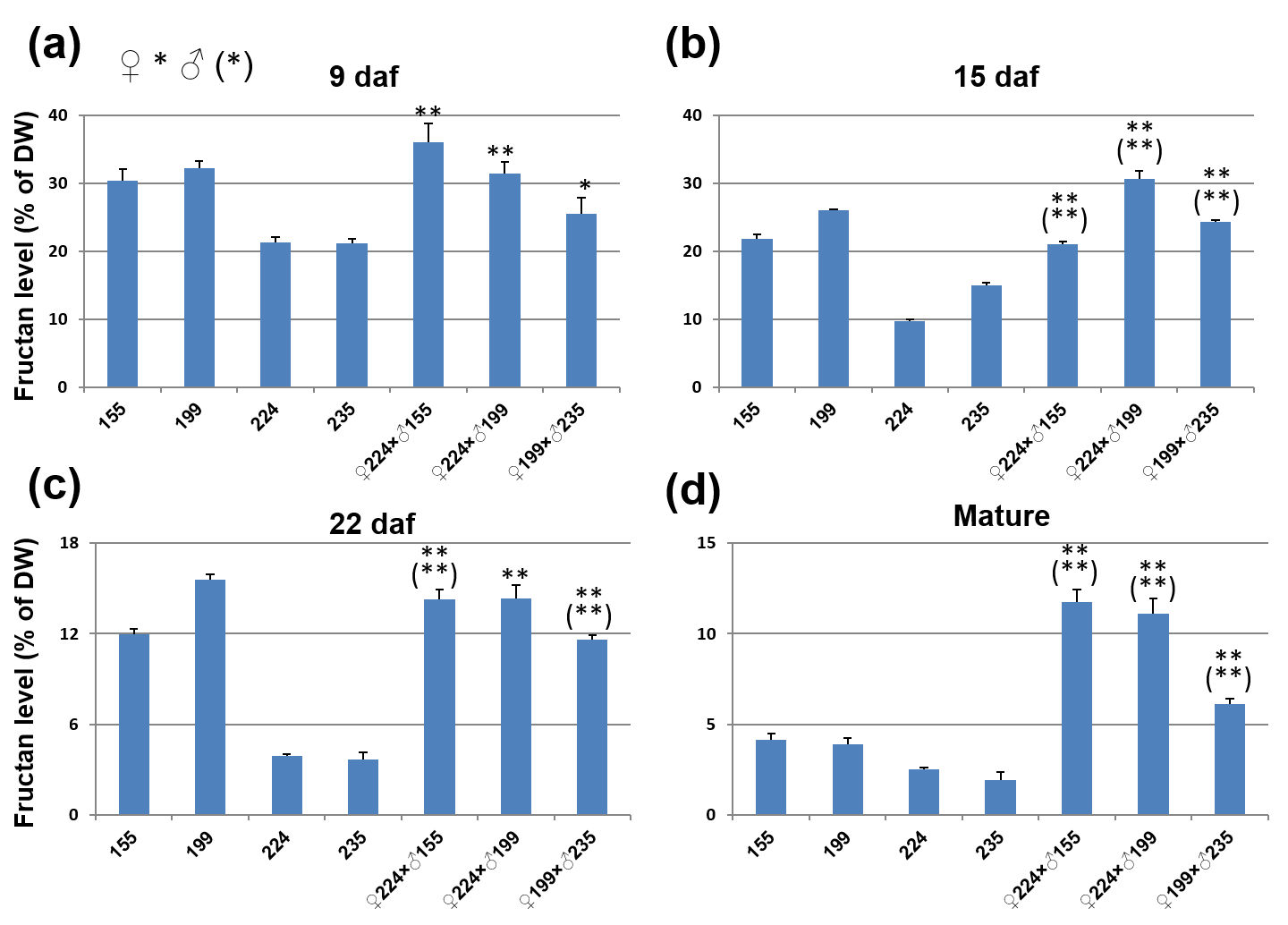
**

**Fig. S3** Fructan percentage per unit dry weight (DW) during grain development at 9 (**a**), 15 (**b**), and 22 (**c**) days after flowering (daf) and at grain maturation (**d**) for G2 (155), G1 (199), and G3 (224 and 235), and their respective F_3_ progenies G3 (224) x G2 (155), G3 (224) x G1 (199), G1 (199) x G3 (235). Student’s t-test was used (Error bars show SD). **P* < 0.05 and ***P* < 0.01 or (*) *P* < 0.05 and (**) *P* < 0.01 are shown for significant differences between the progenies and the maternal or paternal line, respectively. Mid-spike grains from three independent plants (*n* = 3) were used for fructan determination.

**
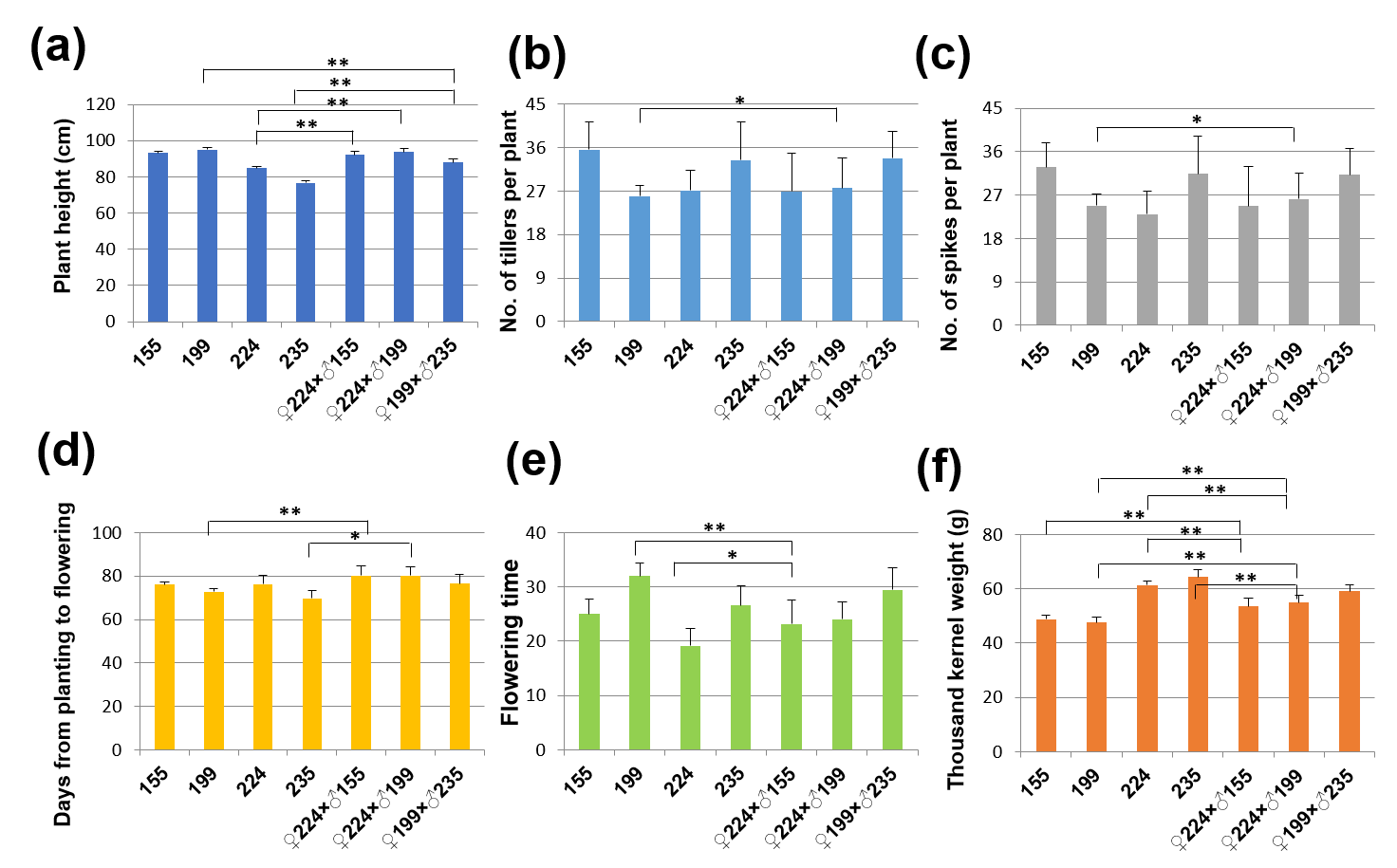
**

**Fig. S4 Phenotyping of F3 progenies in phytotron studies**. **a** Plant height. **b** Number of tillers per plant. **c** Number of spikes per plant. **d** Days from planting to flowering. **e** Flowering time/period (from the first flowering spike to the last flowering spike). **f** Thousand kernel weight. Student’s t-test was used (Error bars show SD). **P* < 0.05 and ***P* < 0.01 are shown for significant differences between progenies and their parents. Five independent plants for parents (*n* = 5) and 10 plants for progenies (*n* = 10) were used for phenotyping.

**
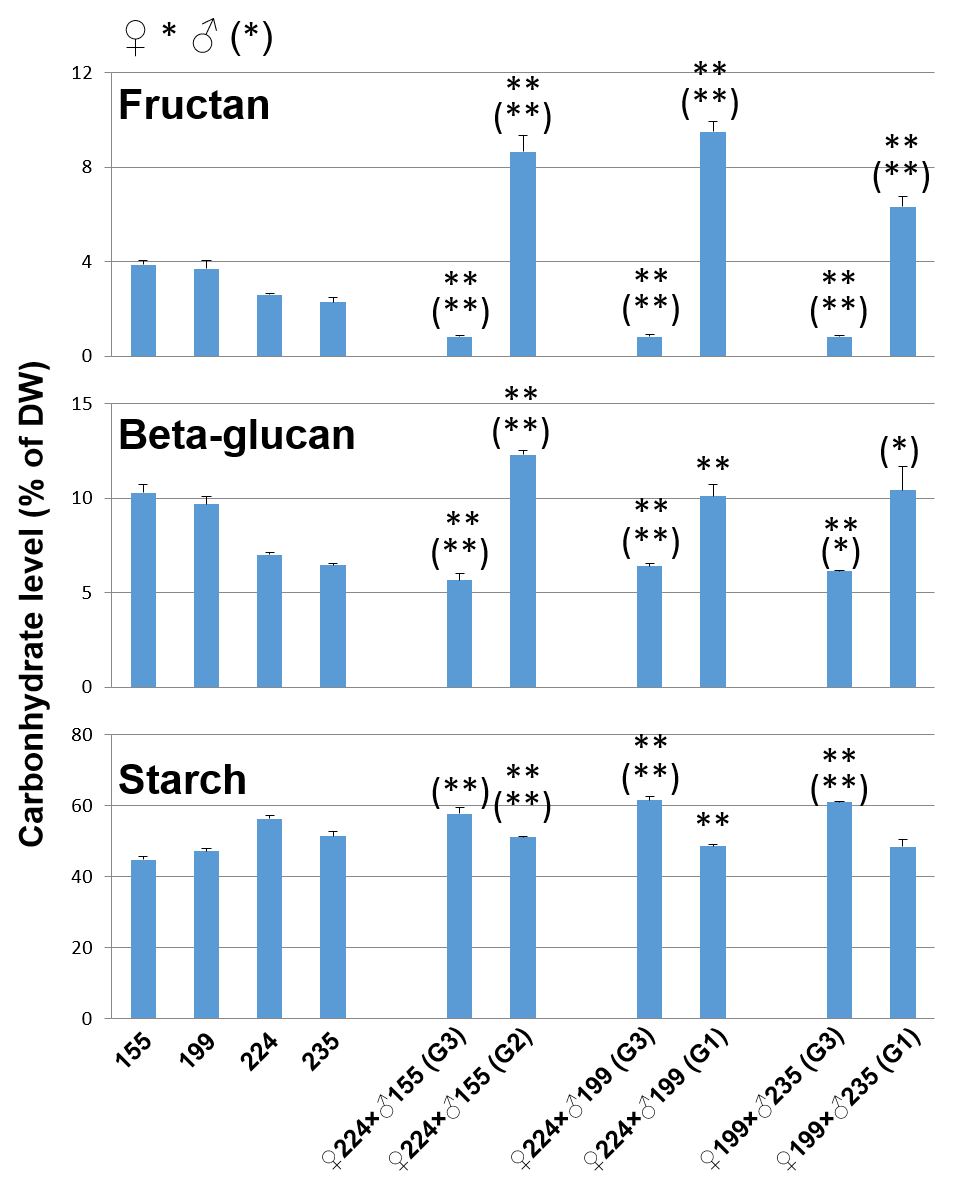
**

**Fig. S5 Percentage per unit dry weight of fructan, beta-glucan, and starch in G1/G2 or G3 shape-inherited grains**. A correlation between fructan and beta-glucan content in all the different line was found (Pearson correlation coefficient (*r*)=0.8698). Student’s t-test was used (Error bars show SD). **P* < 0.05 and ***P* < 0.01 or (*) *P* < 0.05 and (**) *P* < 0.01 are shown for significant differences between the progenies and the maternal or paternal line, respectively. Three biological replicates or grains from three independent plants (*n* = 3) were used for carbohydrate analyses.

**
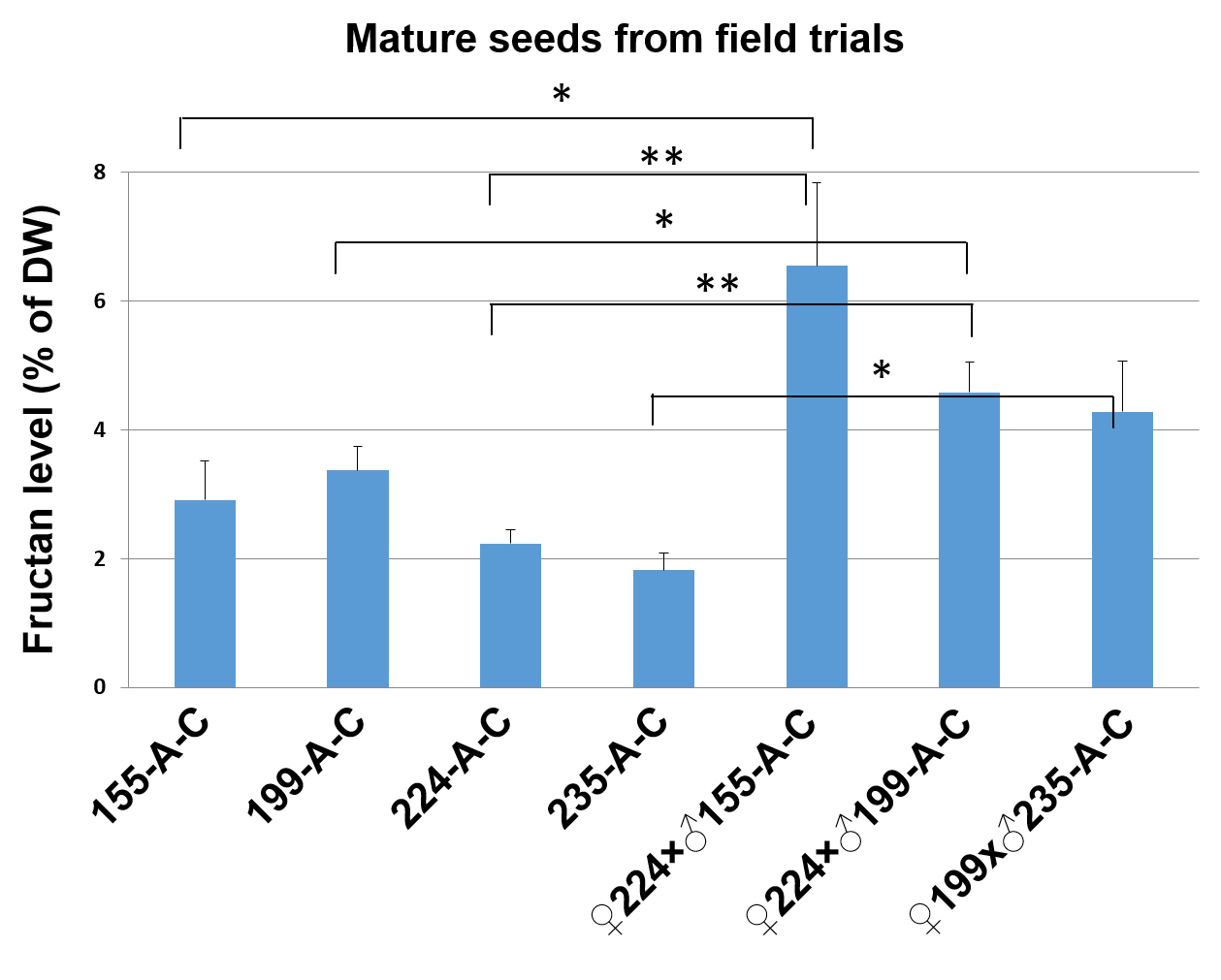
**

**Fig. S6 Field trial on high grain fructan barley in Uppsala.** Three replicates (replicates A-C) of each barley line were randomly distributed in four blocks. Ten plants (1-10) were cultivated for each replicate. Student’s t-test was used (Error bars show SD). **P* < 0.05 and ***P* < 0.01 are shown for significant differences between progenies and their parents. Fructan percentage per unit dry weight (DW) in harvested grain of the high grain fructan barley is average of 3 biological replicates or plots A-C (*n* = 3). Three independent plants in each biological replicate or plot were used.


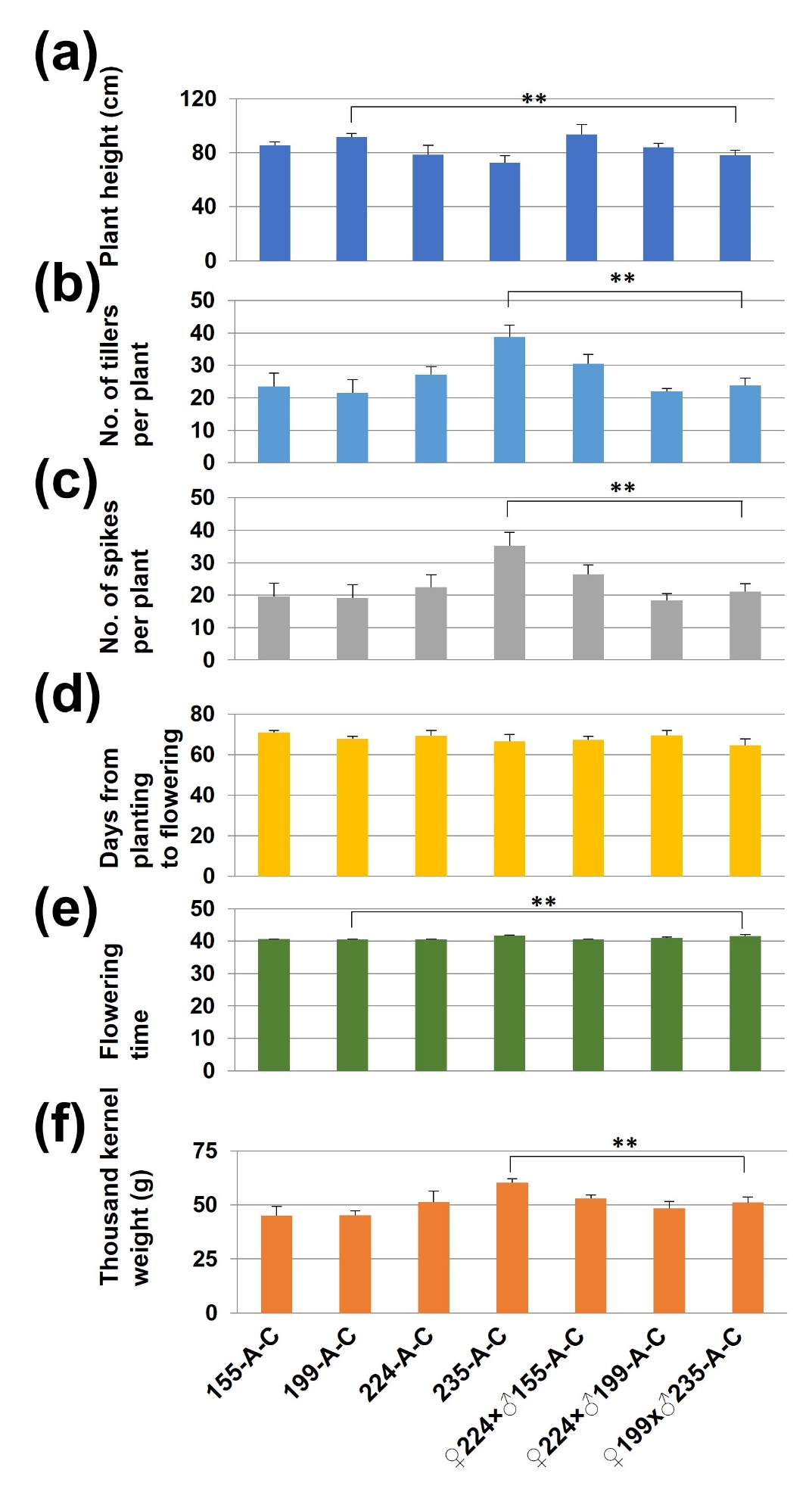


**Fig. S7 Phenotyping of the high grain fructan barley grown in a field trial in Uppsala**. **a** Plant height. **b** Number of tillers per plant. **c** Number of spikes per plant. **d** Days from planting to flowering. **e** Flowering time/period (from the first flowering spike to the last flowering spike). **f** Thousand kernel weight. Student’s t-test was used (error bars show SD). **P* < 0.05 and ***P* < 0.01 are shown for significant differences between progenies and their parents. Data are average of 3 biological replicates or plots A-C (*n* = 3). Ten plants were randomly selected in each biological replicate or plot. A few of the 10 plants in each plot (A, B or C) were damaged by natural conditions.


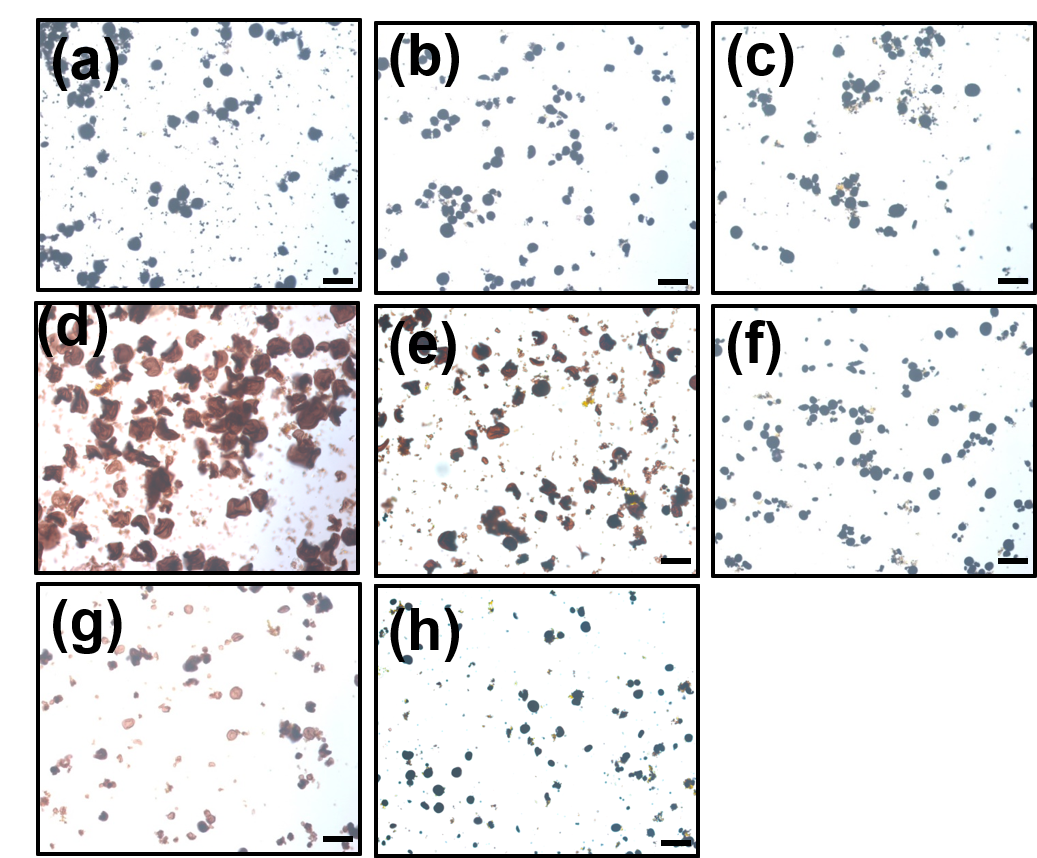


**Fig. S8** Light microscope image of starch granules stained with an iodine solution. **a** Variety 249 or Gustav. **b** 155. **c** 199. **d** 224. **e** 235. **f** 224x155. **g** 224x199. **h** 199x235. Bars = 30 µM.


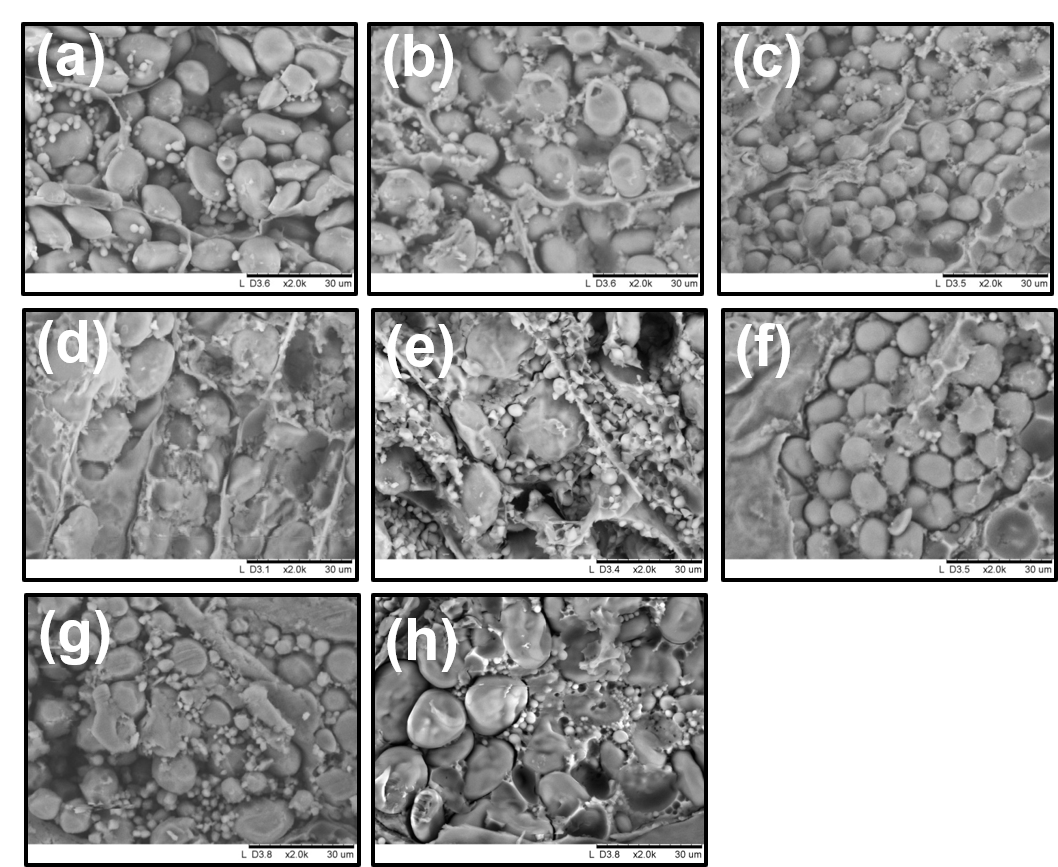


**Fig. S9** Scanning electron micrographs of starch granules from different barley. **a** 249 or Gustav. **b** 155. **c** 199. **d** 224. **e** 235. **f** 224x155. **g** 224x199. **h** 199x235. Bars = 30 µM.


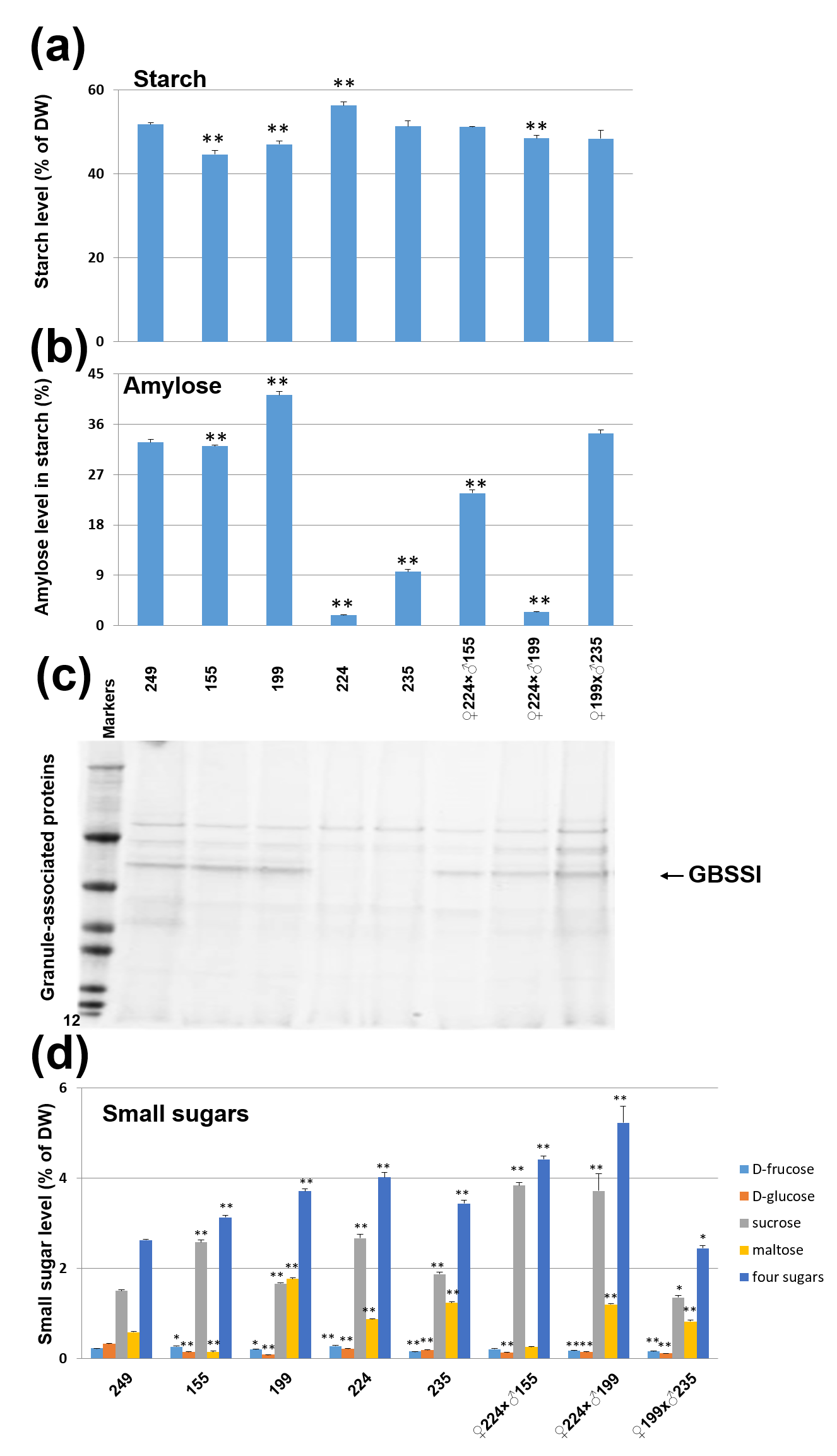


**Fig. S10** Determination of total starch, amylose, granule associated proteins (GBSSI) and small sugars in seeds of high grain fructan barley. **a** Total starch. **b** Amylose level in starch. **c** SAS-PAGE of granule associated proteins. **d** Small guars. Variety 249 or Gustav was used as a control. Barley lines are indicated. Three biological replicates were used for starch and small sugar determination and two for amylose and protein determination. 249/Gustav was used as reference. Student’s t-test was used (Error bars show SD). **P* < 0.05 and ***P* < 0.01 in **a, b, d** are shown for significant differences between barley lines and 249/Gustav. Three biological replicates or grains from three independent plants (*n* = 3) were used for analyses.


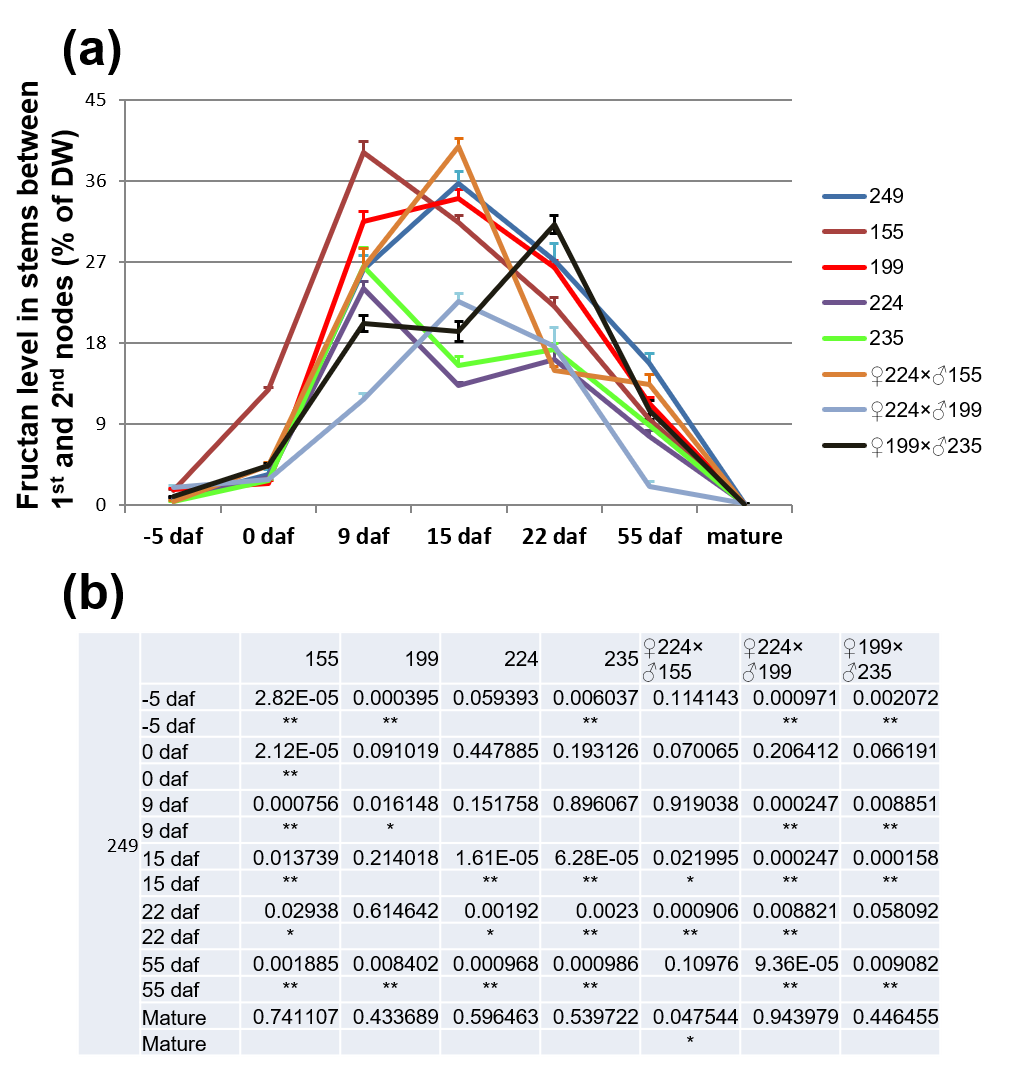


**Fig. S11** Fructan accumulation in stems of penultimate internode before and after flowering in different barley lines from a field trial in Uppsala. **a** Fructan accumulation. **b** Statistical analysis. Student’s t-test was used (Error bars show SD). **P* < 0.05 and ***P* < 0.01 are shown for significant differences between barley lines and 249/Gustav. Three biological replicates or stems from three independent plants (*n* = 3) were used for analyses.

**Table S1. 12 barley lines/cultivars used in this work and their grain fructan levels**

| **Laboratory ID/Line No** | **Line name or Cultivar^a^** | **Fructan level (%)^b^** |
| --- | --- | --- |
| 120 | NGB 114602 | 0.62 (Low) |
| 131 | NGB 20028 | 0.75 (Low) |
| 155 | SLU 7 | 3.90 (High) |
| 181 | KVL 301 | 1.64 (Middle) |
| 198 | KVL 1112 | 3.56 (High) |
| 199 | KVL 1113 | 3.51 (High) |
| 220 | Cinnamon | 1.50 (Middle) |
| 224 | SW 28708 | 2.71 (Middle) |
| 228 | Karmosé | 1.80 (Middle) |
| 235 | SW 49368 | 2.01 (Middle) |
| 236 | SW 49427 | 1.10 (Low) |
| 249 | Gustav | 1.75 (Middle) |

^a^NGB: Nordic Gene Bank or NordGen, Sweden; SLU: The Swedish University of Agricultural Sciences; KVL: The Royal Veterinary and Agricultural University, Denmark; SW: Lantmännen, Sweden. ^b^High: > 1.50%; Middle: ≤ 1.50% and > 1.10%; Low: ≤ 1.10%.

**Table S2. Primers used in this study**

| **Primers for Quantitative PCR** | | | | |
| --- | --- | --- | --- | --- |
| Primer name | Gene name | GenBank Accession No. | Position (nt No.) | Primer sequence(5' 3') |
| SUSIBA1+2F | *HvSUSIBA2* | AY323206 | 1679-1699 | TTGATCTTGGTGTTGGAATCA |
| SUSIBA1+2R |  |  | 1759-1778 | CCCATGGTTTGCATTTGATA |
| SUSIBA2 F | *HvSUSIBA2* | AY323206 | 614-633 | TGAATGAAAACAGACCTCCC |
| SUSIBA2R |  |  | 714-733 | ATGTACCAACTTCAGCAGGC |
| 6-SFTF | *Hv6-SFT* | JQ411254 | 1631-1650 | GACGAACGGCACGACTACTA |
| 6-SFTR |  |  | 1749-1768 | ATAGAAGGAGGTGGACGCAT |
| 1-SSTF | *Hv1-SST* | JQ411252 | 3316-3336 | TCTACCTGTTCAACAATGCCA |
| 1-SSTR |  |  | 3378-3397 | TGGTTGTACGACGAGTCCAT |
| 1-FEHF | *Hv 1-FEH* | AJ605333 | 1641-1659 | GCGGTGGAGAGCTTCGGTG |
| 1-FEHR |  |  | 1763-1780 | GCGCTGAGCTGTGGCACC |
| 6-FEHF | *Hv 6-FEH* | AK357958 | 1689-1706 | TGTACCCTGAGCACGCCG |
| 6-FEHR |  |  | 1771-1788 | GCCAGCCTCCATGCTTCG |
| UbiquitinF | *HvUbiquitin* | AK357664 | 330-351 | GCCAAGAAGCGCAAGAAGAAGA |
| UbiquitinR |  |  | 469-516 | GGTCGAAGTGGTTGGCCATGA |
